# Skin-Inspired Ultra-Linear Flexible Iontronic Pressure Sensors for Wearable Musculoskeletal Monitoring

**DOI:** 10.1007/s40820-025-01887-x

**Published:** 2025-09-01

**Authors:** Pei Li, Shipan Lang, Lei Xie, Yong Zhang, Xin Gou, Chao Zhang, Chenhui Dong, Chunbao Li, Jun Yang

**Affiliations:** 1 Chongqing Institute of Green and Intelligent Technology, Chinese Academy of Sciences, Chongqing, 400714 People’s Republic of China; 2 Department of Orthopedics, the Fourth medical center of Chinese PLA General Hospital, Beijing, 100039 People’s Republic of China; 3 Key Laboratory of Optoelectronic Technology & Systems (Education Ministry of China), Chongqing University, Chongqing, 400044 People’s Republic of China

**Keywords:** Iontronic sensor, Skin-inspired design, Linear range, Linear sensing factor, Biomechanical monitoring

## Abstract

The growing prevalence of exercise-induced tibial stress fractures demands wearable sensors capable of monitoring dynamic musculoskeletal loads with medical-grade precision. While flexible pressure-sensing insoles show clinical potential, their development has been hindered by the intrinsic trade-off between high sensitivity and full-range linearity (*R*^2^ > 0.99 up to 1 MPa) in conventional designs. Inspired by the tactile sensing mechanism of human skin, where dermal stratification enables wide-range pressure adaptation and ion-channel-regulated signaling maintains linear electrical responses, we developed a dual-mechanism flexible iontronic pressure sensor (FIPS). This innovative design synergistically combines two bioinspired components: interdigitated fabric microstructures enabling pressure-proportional contact area expansion (∝ *P*^1/3^) and iontronic film facilitating self-adaptive ion concentration modulation (∝ *P*^2/3^), which together generate a linear capacitance-pressure response (*C* ∝ *P*). The FIPS achieves breakthrough performance: 242 kPa^−1^ sensitivity with 0.997 linearity across 0–1 MPa, yielding a record linear sensing factor (LSF = 242,000). The design is validated across various substrates and ionic materials, demonstrating its versatility. Finally, the FIPS-driven design enables a smart insole demonstrating 1.8% error in tibial load assessment during gait analysis, outperforming nonlinear counterparts (6.5% error) in early fracture-risk prediction. The biomimetic design framework establishes a universal approach for developing high-performance linear sensors, establishing generalized principles for medical-grade wearable devices.

**Graphical Abstract:**

**Supplementary Information:**

The online version contains supplementary material available at 10.1007/s40820-025-01887-x.

## Highlights

- Bioinspired dual-mechanism sensor combining fabric microstructures (∝ *P*^1/3^ contact area) and ionic film (∝ *P*^2/3^ ion modulation) achieves 242 kPa^−1^ sensitivity with 0.997 linearity (0–1 MPa), yielding record LSF of 242,000.
- Medical-grade validation via smart insole demonstrates 1.8% GRF error (vs. 6.5% in nonlinear sensors), enabling precise early fracture-risk prediction and validating medical-grade wearables.

**Supplementary Information:**

The online version contains supplementary material available at 10.1007/s40820-025-01887-x.

## Graphical abstract

**Figure:**
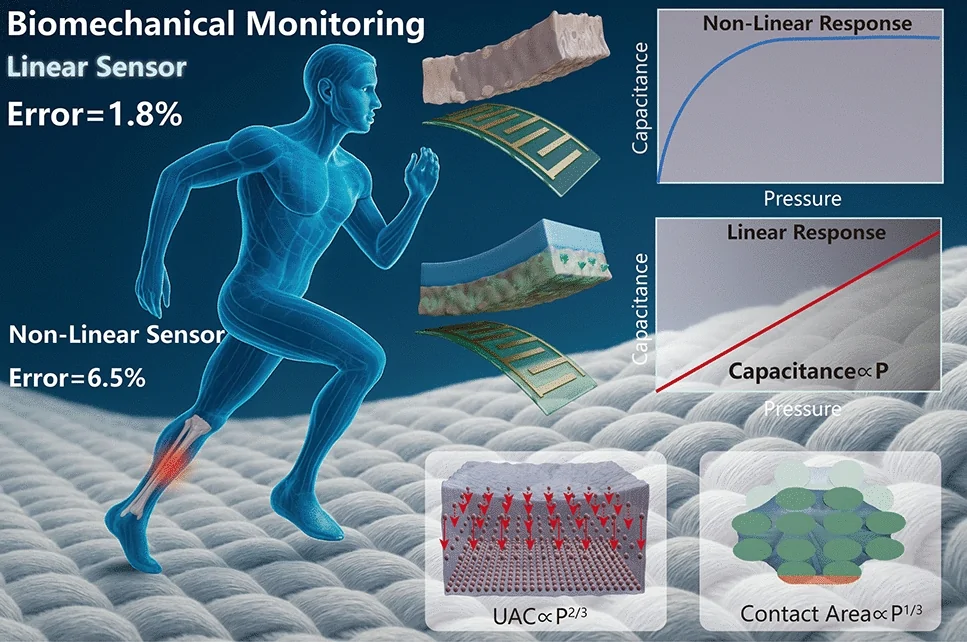

## Introduction

Exercise-induced bone stress injuries (BSI), particularly tibial fractures caused by repetitive mechanical loading during running (accounting for 6–20% of sports injuries [1]), necessitate real-time tibia load monitoring for injury prevention and rehabilitation planning. Current methods—such as invasive strain gauges or costly optical motion capture systems—lack practicality for free-motion monitoring due to irreversible tissue damage or limited portability. While biomechanical platforms like force plates with optical markers remain the gold standard for indirect tibial load estimation, their operational complexity and inability to provide real-time data drive urgent demand for wearable solutions. Flexible pressure sensors integrated into ergonomic devices [2], such as insoles, offer promising alternatives for continuous, non-restrictive tibial load tracking [3–5]. Since monitoring bone loads requires simultaneous acquisition of mechanical and gait-related kinematic data [6, 7], flexible pressure-sensing insoles have emerged as a viable wearable option. To accurately reflect plantar pressure dynamics and achieve simplified signal decoupling in wearable devices, flexible sensors must fulfill stringent requirements: ultra-wide working range, high sensitivity, and full-range linearity [8–10]. Although significant breakthroughs have been achieved in individual performance metrics—such as spatial resolution and response speed—its practical applications still face a series of challenges. Taking pressure sensing for biomechanical monitoring as an example, rigid sensors exhibit a nonlinearity error of ≤ 0.1% F.S., whereas existing flexible sensors typically show a nonlinearity error of ≥ 5% F.S. or adopt segmented linearity over a wide range. This leads to an exponential increase in calibration complexity for flexible sensors, severely limiting both their precision and the reliability of data acquisition, and fundamentally constraining their engineering application in wearable biomechanical monitoring. To characterize the linear response of sensors across a wide range, recent studies have proposed the linear sensing factor (LSF, sensitivity × linear range) = as a critical metric for evaluating sensor linearity [11–13]. Flexible sensors, which employ various response mechanisms such as piezoresistive, piezoelectric, and capacitive, have gained significant attention in recent years. Ionic gel materials, thanks to their ultra-high intrinsic capacitance density (> 1 μF cm^−2^) formed at the electric double-layer interface [13], have shown distinct advantages. Related studies [14–16] confirm that ionic gels can achieve MPa-level large-range sensing while maintaining high sensitivity, making them ideal materials for fabricating high-LSF flexible sensors.

Once materials are selected, structural design becomes a necessary step to further enhance LSF. For instance, introducing internal porous architectures like sponges [17–19] and foams provide expanded deformation space, improving linearity. Similarly, multilayer structures [20–26] mitigate contact saturation by distributing stress across layers, thereby extending the linear elastic range. However, these approaches face inherent limitations: intricate fabrication of porous/multilayer geometries, complex force-electricity coupling behaviors, and challenges in precise modeling for performance optimization. Designing surface microstructures at the material-electrode interface offers a more controllable pathway to stabilize contact area evolution under pressure. Surface topologies such as pyramids [27], micro-domes [28], wave structures [29], and Gaussian curves [30] enable predictable contact mechanics. Yet, single-scale periodic microstructures exhibit limited deformation ranges and rapid stress saturation, limiting sensor’s linearity. To address this, multiscale microstructures—including parallel designs [31–33] (large-scale features incorporating smaller microcolumns/spheres) or serial arrangements [34, 35] (pyramids/micro-domes of varying heights)—have been explored. However, the inherent nonlinear elastic deformation range of flexible substrates, along with the complex and difficult-to-model multi-stage, multilayer deformation mechanisms of flexible materials, continues to limit the extension of the sensor’s linear range. These challenges underscore the need for approaches that move beyond traditional deformation design mechanisms to improve the performance of flexible sensors. It is essential to explore novel sensing mechanisms rooted in the fundamental principles of the sensor, aiming to enhance its linear response, while ensuring that these mechanisms are easily modelable to facilitate further optimization of linear performance.

Inspired by the gradient material architecture of human skin [36–38] (composed of collagen/elastic fibers and matrix), we developed an flexible iontronic pressure sensor (FIPS) integrating fabric and ionic gel. The fabric network, with its distinct Young’s modulus, prevents ionic gel hardening under high pressure, enabling wide-range responsiveness. A theoretical model was established to elucidate the dual mechanisms governing linear sensitivity: pressure-induced contact area expansion (∝ *P*^1/3^) and ion concentration redistribution (∝ *P*^2/3^). The synergistic interplay between these nonlinear factors produces a linear capacitance-pressure response (*C* ∝ *P*). Leveraging this principle, the sensor achieves a sensitivity of 242 kPa^−1^, full-range linearity (*R*^2^ = 0.997) over 1 MPa, a LSF of 242,000. The design strategy is validated across various substrates and ionic materials, demonstrating its versatility. Further, wearable plantar pressure monitoring systems based on this sensor demonstrate experimental validation for tibial load assessment, showcasing its potential in sports biomechanics.

## Mechanism and Design of the Linear FIPS

**Fig. 1:**
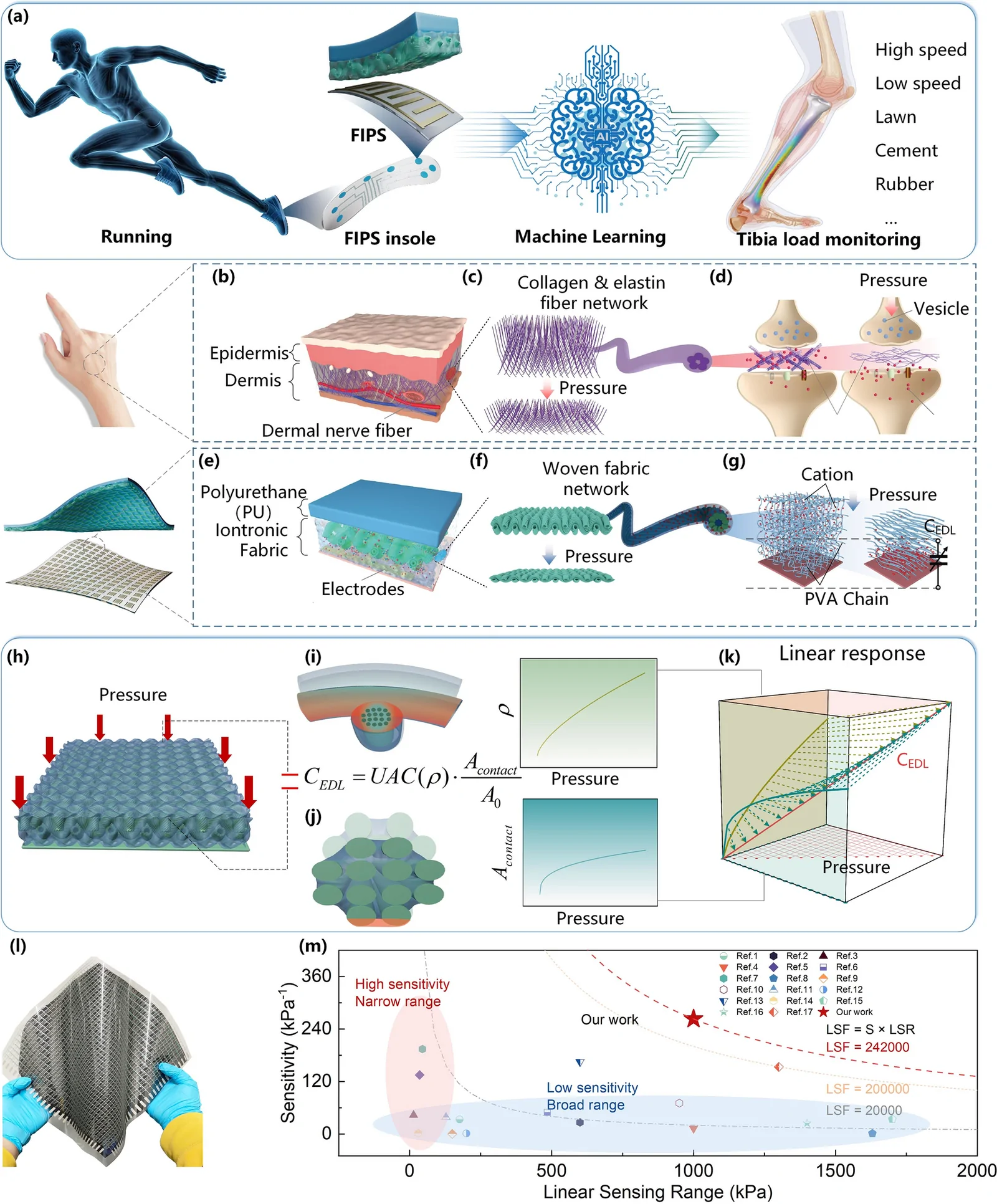
**a** Overall research approach of this article. **b** Structure of the epidermis and dermis of the human skin. **c** Collagen fibers and elastic fiber network in the dermis. The fiber network undergoes deformation under pressure. **d** From a microscopic perspective, collagen fibers unlock under pressure, reducing hindrance to ion transport. **e** Composition of the FIPS, consisting of a PU surface layer, iontronic fabric layer, and bottom interdigitated electrodes. **f** Deformation of the iontronic fabric layer under pressure. **g** Unlocking of PVA polymer chains under pressure facilitates smoother ion migration and an increase in double-layer capacitance. **h** Schematic of applying pressure to the iontronic fabric. **i** Illustration of changing volumetric ion concentration in the ion gel due to pressure on the interwoven iontronic fiber bundle and a representation of volumetric ion concentration over time. **j** Increase in the contact area between the iontronic fiber bundle and electrodes under pressure, with a representation of contact area changes over time. **k** Combining nonlinear changes in volumetric ion concentration and contact area to achieve linear capacitance variation. **l** Composition of a large array FIPS, including the epidermal layer, iontronic fabric layer, and bottom interdigitated electrode layer. **m** The fabricated iontronic sensor demonstrates a LSF of 242,000, the highest value reported to date

Figure 1a presents the conceptual framework of this work. We developed a wearable tibial load monitoring system through a fully integrated design combining a full-scale linear flexible sensor and a customized insole, enabling quantitative assessment of tibial load variations across diverse terrains and running velocities. The human skin serves not only to perceive a wide range of mechanical stimuli but also to protect internal tissues, owing to its structural composition. Comprising the epidermis and dermis, the skin's dermal layer contains a matrix and an elastic fiber-collagen fiber network, as depicted in Fig. 1b, c. Under pressure, the collagen–elastic fiber network compresses, absorbing a significant amount of energy, as illustrated in Fig. 1d. Furthermore, when magnifying a single collagen fiber, as shown in Fig. 1e, the large collagen fiber molecules unlock under pressure, facilitating the transmission of pressure signals to the receptors. Inspired by this gradient material fiber network, we designed the FIPS, a sensor composed of polyurethane and iontronic fabric, with interdigitated electrodes placed at the bottom to transmit electrical signals, as presented in Fig. 1e–g. Similar to the collagen–elastic fiber network in the skin, the woven iontronic fabric network in Fig. 1h bears most of the mechanical stimuli, providing the sensor with a large measurement range. Simultaneously, the contact area between the iontronic fabric and the electrodes increases. Additionally, as shown in Fig. 1i, the iontronic fabric network experiences an unlocking of PVA macromolecules, which accelerates the movement of ions toward pressure gradients, resulting in changing local ion density. Under the synergistic effects of changing local ion density and contact area, the double-layer capacitance (*C*_EDL_) of the iontronic sensor undergoes alterations. Figure 1j illustrates the iontronic fabric under pressure, according to classical electrochemical theory, the *C*_EDL_ can be described as:

1 $$C = {\mathrm{UAC}}(\rho )\frac{A}{A_0 }$$

To gain a clearer understanding of the microstructure generated by the woven fabric on the surface of iontronic fabric, the sensor’s electrode side is oriented upward in the figure, and the electrodes are subsequently removed. Then, we decompose the iontronic fabric’s response under pressure into two parts: the deformation of interwoven iontronic bundles and the change in the contact area of a single bundle with the electrode. Applying the Hertz contact model, we described the deformation of iontronic bundles, leading to a localized change in ion density, as follows:

2 $$\delta = \lambda \left[ {P^2 \left( {\frac{r_1 + r_2 }{{2r_1 r_2 }}} \right)\left( {\frac{1 - \mu_1^2 }{{E_1 }} + \frac{1 - \mu_2^2 }{{E_2 }}} \right)^2 } \right]^\frac{1}{3} = \lambda P^\frac{2}{3} k_1^\frac{2}{3}$$

where α and λ are related to the geometric dimensions of the bundles. In this analysis, assuming the two interwoven iontronic bundles are of the same size, we have α=0.908, λ=2.080, where *r*_*1*_ and *r*_*2*_ are the radii of the two iontronic fiber bundles, *P* is the applied pressure, *μ* is the Poisson’s ratio, *E* is Young’s modulus, and *k*1 is a constant related to Poisson's ratio and Young's modulus. The derivation of the above equation can be found in the supporting information Text S1. The contact area of a single bundle with the electrode is:

3 $$A_{{\mathrm{contact}}} = l\sqrt {\frac{4r_0 k_2 }{\pi }} \cdot (\alpha^{ - 1} \cdot P^\frac{2}{3} \cdot k_1^\frac{1}{2} )^\frac{1}{2} = l\left[ {\frac{{4r_0 k_2 \alpha^{ - 1} }}{\pi }} \right]^\frac{1}{2} \cdot P^\frac{1}{3} \cdot k_1^\frac{1}{4}$$

Then we get the *C*_EDL_:

4 $$C_{{\mathrm{EDL}}} = {\mathrm{UAC}}(\rho )\frac{A}{A_0 } = \frac{{m\lambda k_1^{\frac{11}{{12}}} \cdot {\mathrm{UAC}}_0 }}{A_0 }\left[ {\frac{{4r_0 k_2 \alpha^{ - 1} }}{\pi }} \right]^\frac{1}{2} P$$

Combining $\lambda_{\operatorname{int} }$: an interfacial conformity coefficient, χ: a fabric compliance factor, and pressure-induced effective modulus change (Note S3), we obtain an overall pressure–capacitance relationship:

5 $$C(P) \propto P \cdot E_{{\mathrm{eff}}}^{ - \frac{1}{3}} \cdot E_1^{ - \frac{2}{3}} \cdot \lambda_{\operatorname{int} }^\frac{1}{3} \cdot \chi^\frac{1}{3}$$

Although both the unit-area capacitance (UAC) and the pressure-induced contact area exhibit intrinsic nonlinearity, their coupled interaction compensates for these deviations, resulting in an emergent linear correlation between the electric C_EDL_ and applied pressure. This analytically derived five-factor model underscores that sensing linearity is governed not solely by the modulus contrast between constituent layers, but also by the efficiency of mechanical stress transfer across the ion gel–fabric interface and the spatial distribution of deformation across the textile substrate. Leveraging the simplicity and scalability of the fabrication process (as illustrated in Fig. S5), FIPS arrays can be readily produced. Figure 1l presents the structural configuration of a representative FIPS array, comprising a top polyurethane (PU) encapsulation layer, a middle iontronic woven fabric layer, and a bottom interdigitated electrode array patterned via screen printing. This implementation further demonstrates the feasibility of constructing entirely leather-based sensor arrays for practical wearable integration. Currently, the linear sensing factor (LSF) serves as a pivotal quantitative metric for evaluating the linearity of flexible pressure sensors. The proposed FIPS achieves an outstanding sensitivity of 242 kPa^−1^ over a 1 MPa pressure range, resulting in an unprecedented LSF of 242,000—representing the highest value reported to date for flexible pressure sensors, as shown in Fig. 1m.

**Fig. 2:**
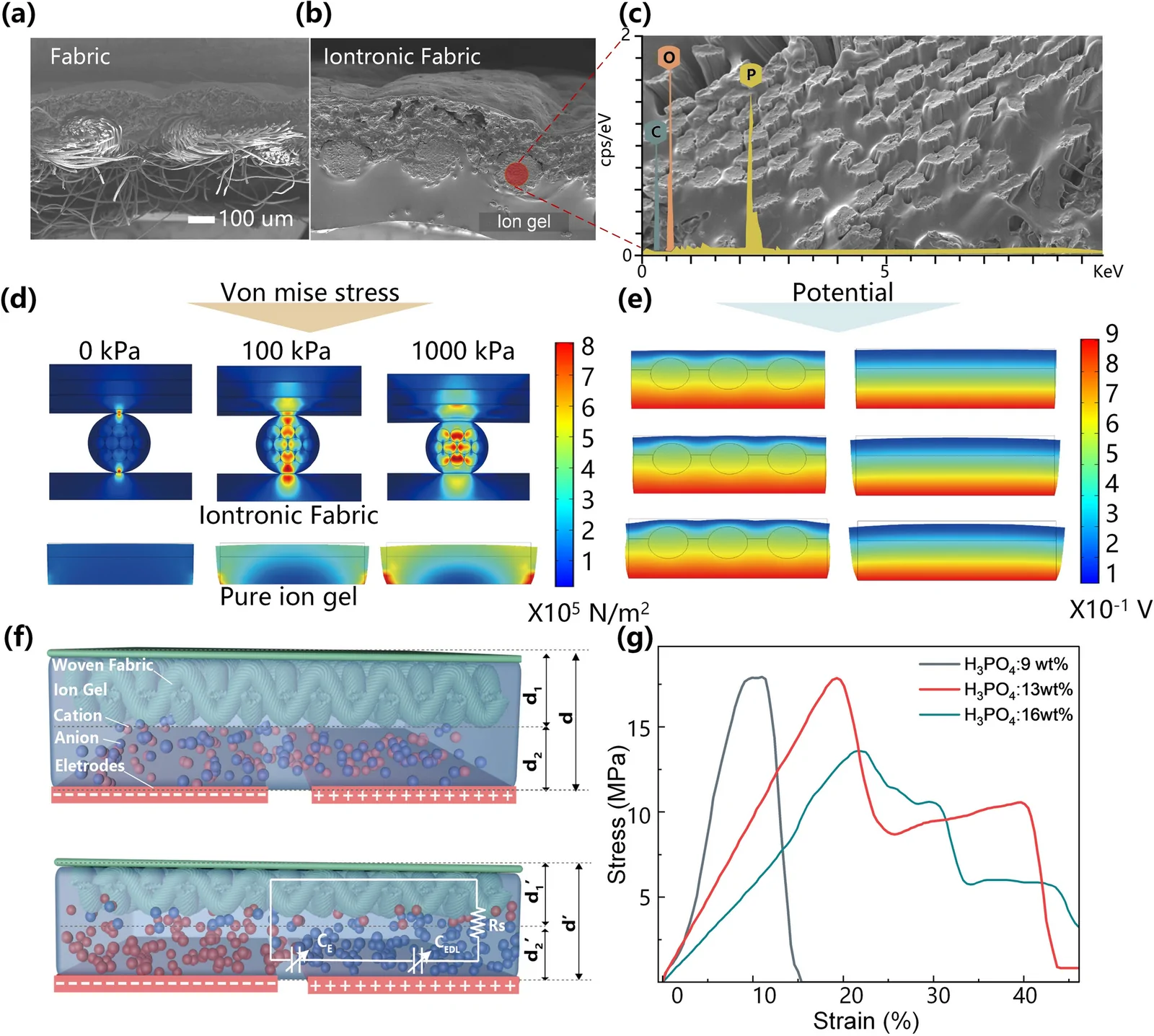
**a** SEM image of the fabric in its natural state. **b** SEM image of the iontronic fabric. **c** Enlarged view of the fiber portion of the iontronic fabric and its component content. **d** Comparison of the equivalent stress between the iontronic fiber bundle and pure ion gel under pressure. **e** Comparison of the electric field distribution between the iontronic fiber bundle and pure ion gel. **f** Schematic illustration of ion migration in the iontronic fabric under pressure. **g** Stress–strain relationship of the iontronic fabric with different phosphoric acid content

Figure 2a displays the SEM cross-sectional image of pure leather fabric, showcasing a weave pattern where fibers intersect, the vertical view of the fabric can be seen in Fig. S6. Pouring ion gel onto the fabric to get the iontronic fabric, with its cross-sectional view depicted in Fig. 2b, and the front SEM image presented in Fig. S7. To confirm the penetration of ion gel into all fabric fibers, we magnified one fiber bundle from the cross section and conducted energy spectrum analysis, the results are shown in Fig. 2c. The presence of abundant phosphorus (*P*) elements in the fibers, a primary component of the ion gel’s phosphoric acid, signifies the ion gel's infiltration into the fiber bundle. Simultaneously, for comparison, the elemental composition of the ion gel was analyzed from a frontal view, as illustrated in Fig. S8. COMSOL analysis was employed to assess the performance of iontronic fabric and pure ion gel under pressure and voltage. To simplify the analysis, the model was reduced to a two-dimensional cross section containing embedded fiber bundles.

Figure 2d describes the Von Mise stress and equivalent strain of iontronic fabric and pure ion gel with embedded fiber bundles under different pressures. It is evident in the pure ion gel that as pressure increases, the gel deformation easily reaches the structural hardening point. However, in contrast to the pure ion gel, the fiber bundles in the iontronic fabric bear the majority of the pressure, preventing the saturation of the ion gel, as reflected in the SEM images of the iontronic fabric under compression in Fig. S9. Additionally, the ion gel beneath the fiber bundles undergoes deformation, leading to an increase in local ion concentration and altering its UAC. Figure 2e illustrates the different voltage distributions under the same potential for iontronic fabric and pure ion gel. The figure shows a distinct localized effect around the fibers, causing increased ion dispersion. Figure 2f includes the essential components of the iontronic sensor: woven fabric, ion gel, cation, anion, and electrodes. The sensor's capacitance change results from the combined action of edge capacitance (*C*_E_) between positive and negative electrodes and the double-layer capacitance (*C*_EDL_) formed between the iontronic material and the electrodes. However, due to *C*_EDL_ being significantly larger than edge capacitance *C*_E_, the edge capacitance effects are neglected in the model. Before compression, the electrodes adsorb a small number of ions. Upon compression, due to changes in the contact area and local ion density, more ions accumulate around the electrodes, causing a significant increase in *C*_EDL_. The content of H_3_PO_4_ affects the stress–strain relationship of the iontronic fabric. In Fig. 2g, guided by previous studies on PVA-based ionic gels, we examined stress–strain curves for H_3_PO_4_ mass fractions of 9, 13, and 16 wt%. We observed that a higher H_3_PO_4_ content results in a lower Young’s modulus for the iontronic fabric, and the yield limit of the fabric increases with the higher phosphoric acid content. This phenomenon may be attributed to the increase in viscoelasticity of the ion gel due to the presence of phosphoric acid. Consequently, we anticipate that the phosphoric acid content will have a significant impact on the linearity and sensitivity of the sensor, and we will delve into this influence in the following sections.

To validate the universality of the proposed design strategy, experiments were conducted using substrates with varying materials, weaving methods, and ion gel systems (PVDF-based). As shown in Fig. S10a, c, leather-textile substrates with plain weave and weft-knitted structures (both integrated with PVDF-based ion gels) were tested. The performance curves of the corresponding iontronic sensors (Fig. S10b, d) demonstrate strong linearity across both configurations. Further, plain-woven fabrics with different thread counts were selected as substrates (Fig. S11a, c). The sensor fabricated from the higher thread count substrate (Fig. S11c) exhibited enhanced linearity compared to the lower thread count counterpart (Fig. S11a), though both maintained robust linear responses. To investigate whether non-cotton fabric substrates could replicate the linearity, glass fiber and nylon textiles were evaluated (Fig. S12). For glass fiber substrates (Fig. S12a, b), poor interfacial bonding between the ionogel and fiber surface resulted in partial exposure of fibers. Additionally, the non-stranded mechanical properties of glass fibers (distinct from cotton) led to reduce linear correlation (*R*^2^ = 0.9715, Fig. S12c). Similarly, nylon substrates (Fig. S12d, e) showed non-conformal iontronic interfaces and suboptimal linearity (*R*^2^ = 0.9787, Fig. S12f). These results confirm that cotton fabrics, with their stranded warp-weft structure and superior compatibility with ion gels, aligned with the derivations in Text S1, are more suitable for constructing wide-range, high-linearity flexible sensors.

## Optimization and Characterization of the FIPS Sensor

**Fig. 3:**
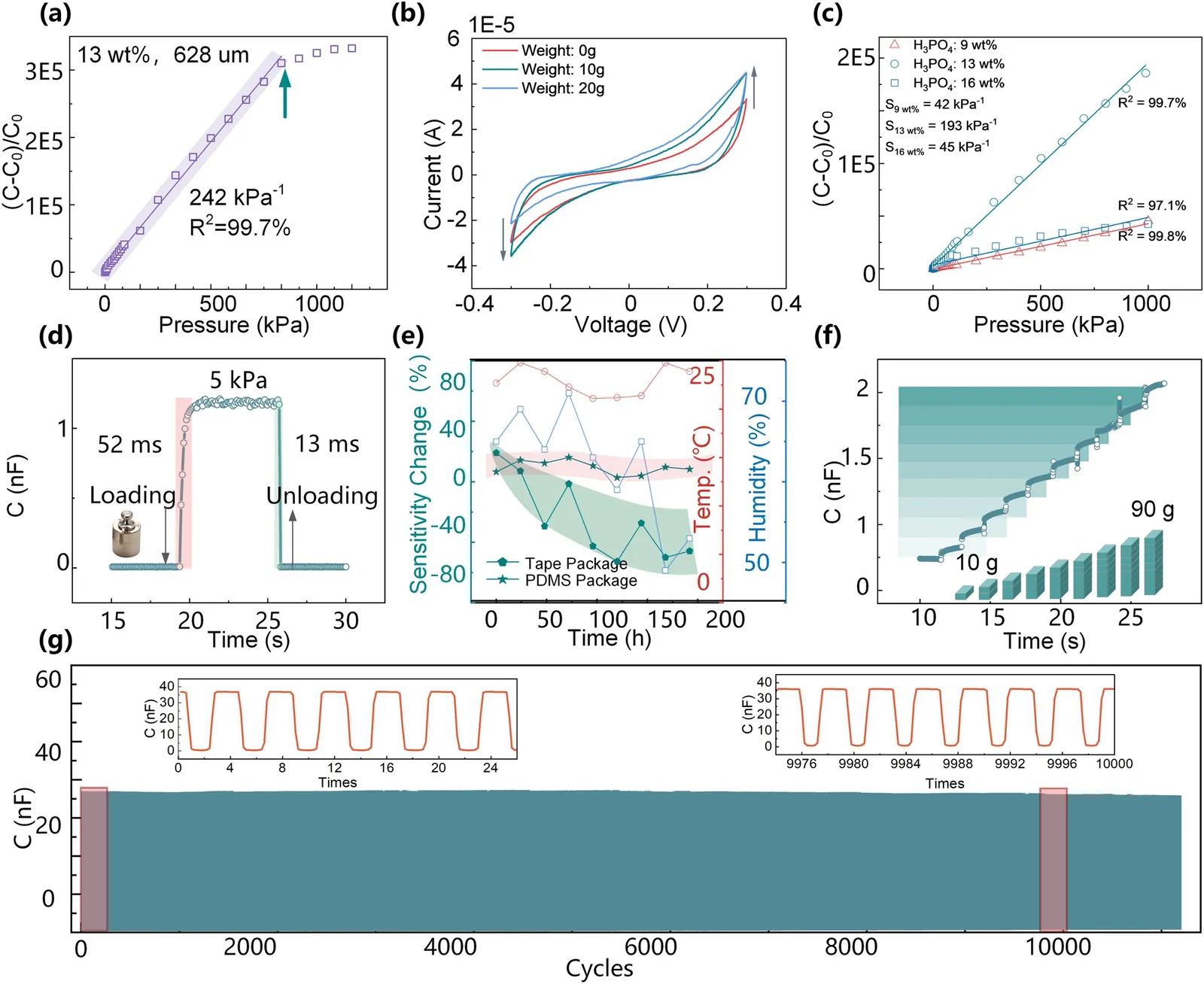
**a** Performance of iontronic fabrics with 628 μm and a phosphoric acid content of 13 wt%. **b** Cyclic voltammetry curves of the iontronic sensor under pressure at different weights. **c** Performance curves of ion gels with varying phosphoric acid content. **d** Response/release times of the FIPS under 5 kPa pressure were measured at 52 and 13 ms, respectively. **e** Sensitivity variations of the sensor under different encapsulation methods to environmental temperature/humidity and their time stability. **f** Linear incremental response of the sensor under linearly increasing pressure. **g** Performance stability of the sensor in over 10,000 repeated load cycles with no significant decline

The thickness of the iontronic fabric also affects the performance of the sensor. We tested sensors of different thicknesses, with consistent H_3_PO_4_ content (13 wt%), in succession and obtained varying sensitivities and linearity, as shown in Fig. S13. Experiments show that the sensitivity of the sensor increases with its thickness initially and then decreases, as shown in Fig. S14. As the thickness increases, the linearity of the sensor decreases. When the sensor thickness reaches 894 μm, its linearity has decreased to *R*^2^ = 86.1%. This indicates that the increase in thickness is primarily due to the thickening of the ionic gel layer. In iontronic fabric with a lower ion gel content, there’s minimal gel thickness on the fabric surface. As the ion gel content increases, the thickness of the ion gel layer in iontronic fabric also rises. It can be easily inferred that iontronic fabric with higher ion gel content exhibits increased sensitivity due to the heightened ion concentration. Regarding linearity, the fiber structure contributes significantly. Thus, as the ion gel content rises, the fiber structure’s contribution diminishes, resulting in a decline in sensor’s linearity. However, if the ion gel content continues to grow, the fiber structure’s contribution to the iontronic fabric becomes nearly negligible. This leads to a decrease in both the sensor’s sensitivity and linearity. This is because the thicker the iontronic fabric, the thicker the pure ion gel layer in the sensor—consequently, the viscoelasticity of the sensor changes, leading to a decrease in linearity. Moreover, we also investigated the influence of interdigital electrode spacing on the performance of iontronic sensors in Fig. S15. We studied the performance of sensors made with interdigital electrodes at spacings of 100, 200, and 500 μm. We observed a distinctive pattern in the influence of the interdigital electrode on sensitivity, wherein sensitivity initially increases and then decreases with the electrode spacing. At a spacing of 200 μm, the sensitivity reaches its peak at 242 kPa^−1^, as illustrated in Fig. 3a. The sensor exhibited full-range linearity within 1 MPa, but after reaching the 1 MPa indicated by the arrow, the input–output curve of the sensor reached a turning point and experienced a linear decline.

Figure 3b presents the cyclic voltammetry scan curves of the sensor under different pressures. It can be observed that under pressure, the redox peaks of the sensor underwent significant changes. This indicated that under the influence of pressure, there's a greater ion migration in the sensor, leading to a substantial increase in the local ion concentration. Figures 3c and S16 illustrate variations in the sensitivity characteristic curve of the FIPS with different H_3_PO_4_ contents—specifically in Fig. 3c (9, 13, and 16 wt%). This non-monotonic behavior can be attributed to H_3_PO_4_ content's impact on two crucial sensor parameters. Firstly, it affects ion concentration, influencing both the initial and final capacitance values. A higher H_3_PO_4_ content leads to a larger initial capacitance, causing a shift in the final capacitance of the sensor. Specifically, the FIPS sensitivity was 42 kPa^−1^ with 9 wt% H_3_PO_4_ 193 kPa^−1^ with 13 wt%, and 45 kPa^−1^ with 16 wt%. Notably, the linearity of FIPS with 9 wt% H_3_PO_4_ was *R*^2^ = 99.8%, whereas those with 13 and 16 wt% phosphoric acid were *R*^2^ = 99.7% and *R*^2^ = 97.1%, respectively. The increase in H_3_PO_4_ content resulted in a decrease in material elasticity, proportional to the decrease in the sensor's linearity. Figure S16 confirms that the optimal performance trade-off occurs at approximately 13 wt% H_3_PO_4_, which balances sufficient ionic mobility with mechanical stiffness to support stable deformation and ion distribution. Figure 3d presents the response time of the iontronic fabric sensor. Under a 5 kPa step signal, the sensor's signal rose within 52 ms, and after pressure removal, the sensor's signal recovered to the baseline within 13 ms. Figure S16 presents the sensing performance of FIPSs produced in the same batch. The three sensors exhibited excellent full-range linearity and high sensitivity. The sensitivities were measured as 232, 231, and 229 kPa^−1^, while the linearity was 99.4%, 99.4%, and 99.7%, respectively. This revealed a sensitivity error of 0.69% among the three sensors, indicating that the FIPSs fabricated in this paper not only have a simple manufacturing process but also demonstrate good consistency. Figure S17 depicts the hysteresis characteristic curve of the sensor. The blue color gradient from dark to light indicated the number of loading cycles the sensor underwent. The hysteresis characteristic of the sensor is tested after 0, 250, 500, 750, and 1000 cycles, respectively. As shown in the figure, the hysteresis characteristics of the sensor improved after multiple compression cycles, but hysteresis cannot be eliminated, which relates to the sensor’s storage modulus. To quantify hysteresis, we evaluated the output deviation during the 1000th loading–unloading cycle. The sensor exhibited a maximum hysteresis of 4.71%, calculated according to the standard hysteresis error formula. This low value confirms good reversibility and stability under repetitive mechanical stimuli.

A sensor requires not only good sensing performance but also excellent stability. However, stability has consistently posed a challenge for iontronic sensors. Figure 3e illustrates the change in sensitivity over time for the sensor under various encapsulation methods (Fig. S18). Using the PVA/H_3_PO_4_ from this study as an example, we previously investigated the time stability of the ionic gel and determined that it is related to the water content of the ion gel. This also holds for iontronic fabric. Considering this issue, we examined the impact of the ionic gel's packaging method on its time stability and fabricated sensors encapsulated with tape and PDMS, sensors encapsulated with TPU tape only and thin ion gel sensors. As seen in the figure, the sensitivity of the sensor packaged with PDMS remains relatively stable and exhibited only slight fluctuations over time, with an absolute value of change not exceeding 10%. The sensor without a PDMS package experiences considerable water loss, which is not evident within the first 100 h, but the sensor's sensitivity declines by more than 40% as time progresses, and is more easily affected by humidity. This demonstrated that fully sealing the sensor with PDMS effectively mitigates the water loss of the ion gel, enhancing its stability. However, the package also impacted the sensor's initial sensitivity, as the PDMS drying process during sensor coverage introduced additional prestress, increasing the sensor's initial capacitance and affecting its sensitivity. Meanwhile, as depicted in Fig. S20. The unencapsulated sensors showed severe performance degradation: over 65% decline in sensitivity, and pronounced signal fluctuation due to uncontrolled moisture loss and ionic imbalance. PU tape-encapsulated sensors exhibited slightly improved stability, but remained susceptible to environmental humidity because of the breathable nature of the medical tape. The PDMS-sealed sensors demonstrated markedly superior stability, with only a ~ 10% decrease in sensitivity over 912 h. Moreover, signal drift was minimal, confirming the effectiveness of PDMS as a humidity and temperature barrier. This demonstrates that while packaging cannot fully eliminate ionic drift over extended timeframes, it can significantly delay and reduce instability, making the sensor far more reliable for long-term or wearable deployment. Figure 3f depicts the response curve of the sensor during the sequential addition of 10 g weights, ranging from 10 to 90 g, on the sensor. The graph illustrates a progressively increasing response from the sensor, showcasing excellent linearity throughout the incremental weight additions. Figure 3g demonstrates the sensor's cycle stability. Under more than 11,000 cycles, the sensor's capacitance value remains stable with no significant changes, indicating good cycle stability.

Additional tests were conducted to evaluate sensor response under bending. The results in Fig. S21 show that bending induces a capacitance increase, primarily due to local contact area growth and ion redistribution, consistent with the EDL sensing mechanism. However, due to limited stretchability of the woven substrate, the sensor is not suitable for tensile strain measurement. These findings confirm that while the sensor is bendable, its primary sensitivity is to pressure-derived deformation.

The FIPS demonstrates an expansive measurement range, remarkable sensitivity, and exceptional linearity, making it suitable for monitoring a wide spectrum of signals. Its capabilities range from capturing subtle signals such as pulse and respiration to effectively measuring more substantial pressures, such as those associated with foot and car tire pressures. The FIPS is capable of measuring weak signals like pulse waveforms, as well as capturing more substantial signals, such as human body weight or the instantaneous impact force from a hammer striking a table, as shown in Fig. S19. In Fig. S20a–f, the sensor's ability to measure small air fluctuations is further demonstrated, capturing the entire damping process of a spring's dissipated energy, as well as heel pressure during jumping. To further validate the sensor's broad measurement range and high resolution, we placed it under a car, as shown in Fig. S20g, and added four water bottles, each weighing 500 g. The results, illustrated in Fig. S20h, show that the sensor can detect subtle pressure changes even under substantial loads, with a high resolution of 0.03%. Another notable feature of the FIPS is its remarkable scalability in fabrication. Its design and structure allow for seamless expansion into large arrays, revealing a wide range of potential applications. As shown in Fig. S20i, j, we successfully created 8 × 8 and 32 × 32 sensor arrays, capturing diverse pressure signal dynamics across the extensive grid. The flexibility in array configurations, combined with strong performance metrics, underscores the versatility of the FIPS.

## FIPS for Wearable Tibia Load Monitoring

**Fig. 4:**
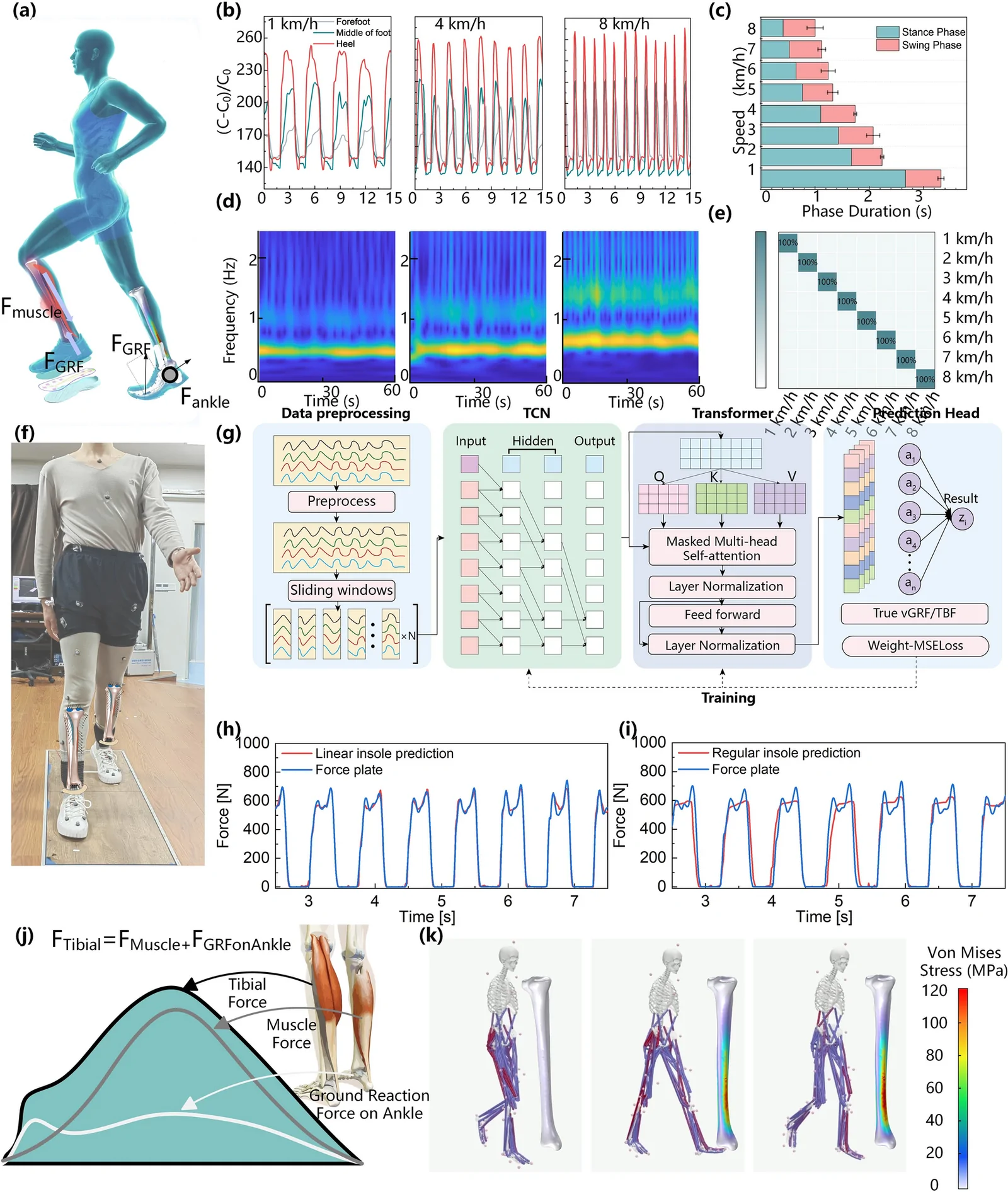
Estimation of tibial load using FIPS insole under laboratory conditions. **a** Schematic diagram of insole-based estimation of ground reaction forces (GRF) and tibial load. **b** Comparison of pressure variations at the forefoot, midfoot, and heel regions during walking at 1, 4, and 8 km h^−1^. **c** Changes in gait cycle duration, stance phase, and swing phase across different walking speeds. **d** Wavelet transform analysis of periodic signals acquired at varying walking speeds, highlighting frequency component variations. **e** Confusion matrix for walking speed classification. **f** A volunteer walking on a force platform with optical motion capture system markers attached. **g** BiLSTM_Attention algorithm for GRF estimation. **h** Comparison of GRF estimated by linear flexible sensors versus force platform measurements. **i** Comparison of GRF estimated by nonlinear flexible sensors versus force platform measurements. **j** Decomposition of tibial forces, primarily comprising muscular forces and ground reaction forces. **k** Finite element simulation results of tibial stress distribution

To further validate the sensor’s applicability in tibial load monitoring, we integrated the FIPS into an insole, enabling real-time monitoring of gait parameters, particularly during high-impact activities such as running, as shown in Fig. 4a, then we conducted a linear calibration of the iontronic insole, as can be seen in Fig. S26. The system consists of a power source, control module, wireless transmission mechanism, and a securing sleeve, with careful attention given to ergonomics and portability in its design, as illustrated in Fig. S21. Our treadmill experiments involved male and female volunteers with regular exercise habits and no sports injuries in the past 3 months. In Fig. S22a, a male volunteer is shown wearing the FIPS insole, with the sensor's control circuit and battery module strapped to his ankle. To minimize discrepancies due to different shoe soles, all volunteers wore experimental shoes from the same manufacturer. Figure S22b outlines the workflow of signal collection by the sensor. This process involves initial data acquisition from both feet, followed by signal processing, which includes outlier detection and replacement. The peak detection algorithms, illustrated in Fig. S23, play a critical role in analyzing the intricate details of the gait cycle, effectively distinguishing between the swing and stance phases.

We collected pressure insole data from male and female volunteers running at speeds of 1–8 km h^−1^, with raw data shown in Figs. S24 and S25. Further analysis, presented in Fig. 4b, c, reveals the dynamic nature of walking. Notably, an increase in walking speed led to a noticeable change in the gait cycle. The cycle’s temporal duration shortened, accompanied by an expansion of the swing phase at the expense of the stance phase. This phenomenon was effectively captured by the FIPS insole sensor. The sensor demonstrated remarkable capability in delineating precise pressure variations across different regions of the foot, including the forefoot, midfoot, and heel. This ability was particularly evident in walking patterns observed at 1 km h^−1^. Additionally, wavelet transform analysis, presented in Fig. 4d, revealed that the frequency of the captured signal increased with walking speed. These nuanced observations serve as a testament to the sensor’s precision and its ability to monitor detailed gait characteristics.

After extracting critical feature values, we used the support vector machine (SVM) algorithm to classify walking speeds accurately (Note S5). A thorough test, involving 10 volunteers walking on a treadmill, was conducted to evaluate the effectiveness of this classification system. The results, captured in the confusion matrix in Fig. 4e, showed an impressive accuracy rate of ~ 100%, highlighting the system’s precision and reinforcing the significant potential of the FIPS insole for applications such as sports performance analysis. To estimate tibial forces, a synchronized acquisition system was established in the laboratory as Fig. 4f. A volunteer wearing tight-fitting clothing with optical motion capture markers walked on a force platform while wearing the FIPS insole, as shown in Video S1, allowing simultaneous collection of force platform signals, motion capture data, and FIPS insole outputs. A bidirectional long short-term memory (BiLSTM) algorithm with attention mechanisms was employed to learn ground reaction forces (GRF) based on the temporal characteristics of the insole pressure signals. To evaluate the performance enhancement of linear sensors, a linear flexible sensor (denoted as Linear Sensor) and a nonlinear counterpart (denoted as Nonlinear Sensor) were compared using identical volunteer data and model parameters. The Linear Sensor dataset comprised 1096 valid samples (80% training, 20% testing), while the Nonlinear Sensor dataset included 1083 samples (80% training, 20% testing). As shown in Fig. 4h, i, the Linear Sensor achieved a GRF estimation error of 1.8%, significantly lower than the 6.5% error of the Nonlinear Sensor, confirming the superiority of linear sensing for GRF prediction, the differences in MAE, RMSE, and R2 of different insole are listed in Table S3. Muscle forces during gait were computed in OpenSim using motion capture marker trajectories (Fig. S26). Since tibial loads primarily originate from GRF and muscle forces, as depicted in Fig. 4j, finite element simulations were conducted to analyze tibial stress distributions in Fig. 4k. Initial Single-Limb Support (Right Leg): Minimal tibial stress due to reduced muscle activation (tibialis anterior, iliopsoas, quadriceps) and absence of GRF during swing phase. Double-Limb Support Transition: Gradual stress increase as GRF and calf muscle activation (soleus, gastrocnemius) intensify during weight shifting. Single-Limb Support (Left Leg): Peak tibial stress occurs with maximal GRF and calf muscle engagement. Late Single-Limb Support: Stress reduction as inertial motion and thigh muscle activation dominate. Axial Von Mises stress distributions along the tibia in Fig. S27 revealed maximum stresses at the distal third of the tibia (0.46 and 0.63 s), corresponding to left-leg single-limb support. Circumferential stress analysis at the distal third as depicted in Fig. S28 showed peak stresses in the coronal plane, consistent with data reported, validating the finite element model.

**Fig. 5:**
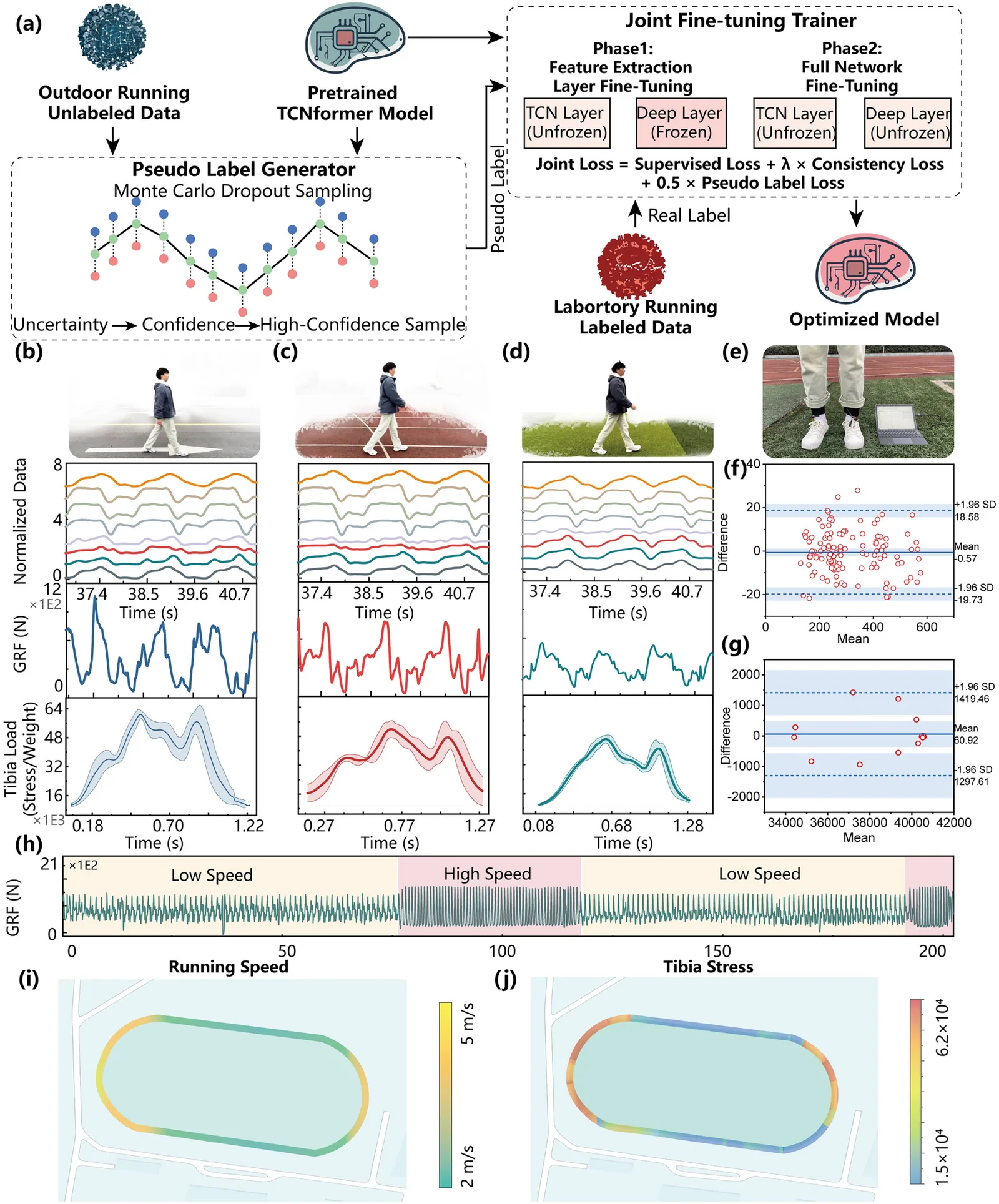
Real-time tibial load monitoring using FIPS insole in outdoor scenarios. **a** Semi-supervised fine-tuning method based on pseudo-label generation and consistency regularization. **b** A volunteer wearing the FIPS insole walking on a concrete floor, raw signals, estimated GRF, and peak tibial stress predictions during concrete floor walking. **c** A volunteer wearing the FIPS insole walking on synthetic track, raw signals, estimated GRF, and peak tibial stress predictions. **d** A volunteer wearing the FIPS insole walking on lawn, raw signals, estimated GRF, and peak tibial stress predictions. **e** Data acquisition system. **f** Consistency analysis of GRF across different gait phases during grass walking. **g** Consistency analysis of peak tibial stress across different gait phases during grass walking. **h** Continuously GRF predictions at varying running speeds. **i** Continuously recorded running speed variations of the volunteer. **j** Peak tibial forces at different running speeds

To further validate the practical application of the FIPS insole for tibial load monitoring under real-world conditions, volunteers wearing the FIPS insole were instructed to walk on lawn (Video S2), plastic tracks (Video S3), and concrete floor (Video S4). The previously trained deep learning model, which predicted tibial loads using laboratory data, demonstrated high prediction accuracy. However, when applied to real-world scenarios, its performance significantly deteriorated. This discrepancy is mainly due to the limited in-place running protocol used in the lab, which differed from the actual running conditions. To address this issue, this section introduces a semi-supervised fine-tuning method based on pseudo-label generation and consistency regularization. This approach effectively enhances the model's adaptability across environments, even in the absence of labeled data from real-world running scenarios, as shown in Fig. 5a. The core idea of the pseudo-label generation and consistency regularization method is to leverage unlabeled real-world running data to improve the model's adaptability to the target domain and to fine-tune the previously trained tibial load estimation model using insole sensor data. The method consists of two main phases: first, high-confidence pseudo-labels are generated using Monte Carlo Dropout; second, laboratory data and pseudo-labeled samples are jointly used for two-phase fine-tuning training.

The raw signals, ground reaction force (GRF) predictions, and estimated tibial stress during walking on different surfaces are presented in Fig. 5b–d, with the data acquisition system shown in Fig. 5e. The rigid and low-damping properties of cement resulted in direct transmission of impact forces during foot–ground contact, yielding significantly higher instantaneous pressure peaks compared to the other surfaces. The synthetic track, with moderate stiffness, attenuated partial impacts, leading to lower pressure amplitudes relative to cement. Conversely, the compliant lawn distributed plantar pressure more diffusely, resulting in the lowest GRF magnitudes. Distinct peak patterns were observed in the signals: walking on cement exhibited dual prominent peaks (during the initial contact and push-off phases) due to the reduced stance time, which was aimed at minimizing discomfort; the synthetic track showed smaller contact-phase peaks relative to push-off peaks; and walking on grass demonstrated smoother GRF transitions owing to dynamic pressure redistribution. Figure 5f confirms the high consistency of GRF across multiple gait cycles on the same surface, validating the reliability of the data. Figure 5g further demonstrates consistent tibial stress distributions during grass walking, with minimal variability between cycles. Continuous GRF predictions during running at varying speeds (Fig. 5h) revealed elevated GRF magnitudes at higher speeds. Correspondingly, Fig. 5i, j illustrates proportional increases in tibial loads with running speed, consistent with biomechanical principles. These results collectively confirm the feasibility of the FIPS insole for biomechanical monitoring in sports medicine applications.

## Conclusions

In conclusion, this study systematically investigates the synergistic coupling between local ion concentration variations and electrode contact area evolution in iontronic pressure sensors under mechanical loading. Theoretical analyses reveal a power-law correlation between local ion concentration and applied pressure (∝ *P*^2/3^), alongside another power-law dependence of contact area on pressure (∝ *P*^1/3^). Through coordinated regulation of these dual mechanisms, we achieve a linear capacitance-pressure response (Δ*C*/*C*₀ ∝ *P*) across the full operational range. This principle enables the fabrication of a flexible pressure sensor exhibiting exceptional performance metrics: ultra-wide detection range (~ 1 MPa), high sensitivity (242 kPa^−1^), and full-range linearity (*R*^2^ > 0.997). The universality of this design strategy is rigorously validated through systematic studies involving diverse substrate materials (cotton, leather), weaving architectures (plain, weft-knitted), and ion gel systems (PVDF-HFP based). Integrated with optical motion capture systems, the linear sensor demonstrates superior GRF prediction accuracy (1.8% error), significantly lower than the 6.5% error observed in nonlinear counterparts, thereby confirming its intrinsic advantage for biomechanical monitoring. Field trials using smart insoles further validate robust tibial load tracking capabilities across heterogeneous terrains (concrete, plastic, lawn). This work establishes a universal paradigm for developing high-performance linear flexible sensors through mechanistic synergy engineering. The demonstrated success in continuous bone load monitoring highlights the transformative potential of linear sensing technology in sports medicine, rehabilitation robotics, and human–machine interfaces.

## Methods

### Preparation of Ion Gel

Poly(vinyl alcohol) (PVA, Mw ~ 145,000, Aladdin Industrial Corporation) and deionized water were mixed at a mass ratio of 1:9. The mixture was vigorously stirred at 90 °C for 2 h to achieve complete dissolution and cross-linking. After cooling to room temperature, phosphoric acid was introduced into the PVA solution under continuous stirring for an additional 2 h to ensure homogeneity. The resulting solution was allowed to stand for 2 h to eliminate residual bubbles entirely. PVDF-HFP powder (Aladdin Industrial Corporation) was dissolved in a mixture of acetone and tetrahydrofuran (THF) under continuous stirring at 50 °C for approximately 4 h until a transparent solution was obtained. Subsequently, 1-ethyl-3-methylimidazolium bis (trifluoromethylsulfonyl) imide ([EMIM][TFSI]) was added to the solution in a predetermined mass ratio, followed by ultrasonic dispersion for 30 min to yield the homogeneous PVDF-based ion gel precursor.

### Preparation of a Dielectric Layer

The dielectric samples produced in this paper are mainly divided into three types: nylon mesh gel, cotton fabric gel and woven polyurethane (PU) leather gel. Nylon mesh contains the mesh number of 20, 30, and 40. Cotton (6536c, 6725e, 6732, 9738) used in this paper is purchased from Tianjin Textile Co., Ltd. The woven PU leather is an artificial leather with woven fabric as a bottom. Three kinds of dielectric layer materials were obtained by dropping ionic gel liquid on nylon mesh, cotton cloth and leather fabric, respectively, and left at room temperature for 48 h.

### Preparation of FIPS Insole

A standardized fabrication protocol was established to ensure batch-to-batch consistency of the ionic flexible pressure-sensing (FIPS) insole. First, the ionogel precursor solution was prepared and uniformly coated onto a leather-textile substrate via doctor-blade coating. The ionically conductive textile was then cut into 10 mm × 10 mm squares to guarantee dimensional uniformity. To ensure encapsulation consistency, laser cutting was employed to fabricate uniformly sized double-sided adhesive spacer layers. These spacer layers were positioned between the ionically conductive textile and the FPC interdigital electrode. The initial capacitance values and sensitivity of the sensing units were measured using an LCR tester to evaluate response consistency. If the consistency met predefined criteria, the encapsulation proceeded; otherwise, the sensing units were reattached and retested until satisfactory consistency was achieved. Following encapsulation, a linen fabric anti-slip layer and a silicone cushion layer were integrated onto the sensor to enhance mechanical stability and wearing comfort.

### Characterization and Measurements

The capacitance changes of the pressure sensor were measured by a TH-2827B LCR meter. Connect the sensor to the TH-2827B LCR meter and measure the change in the sensor's capacitance at a frequency of 10 kHz with a 30 mV AC. Different forces are applied through the force precise control equipment SBA-50S (Jindun Test Equipment Co., Ltd). All tests were carried out using an iontronic pressure sensor with a size of 1 cm^2^. The measurement of the radial artery pulse wave was carried out by attaching the iontronic pressure sensor to the wrist where the pulse could be detected at a testing frequency of 1 kHz. All other capacitance signals were tested at a frequency of 10 kHz unless otherwise specified.

### Multiphysics and Biomechanical Simulations

Multiphysics simulations of the ionic textile's deformation and electric field evolution under pressure were performed using COMSOL Multiphysics®. The Young’s modulus of the ionogel, derived from universal tensile testing machine measurements, was assigned to the material model. The fiber bundle was modeled as a composite structure with an outer annular gel matrix enclosing multiple cylindrical fibers. The model represents a single cross section of the fabric microstructure. Ionic Gel Matrix: Modeled as a hyper-elastic material using the Neo-Hookean model, Young’s modulus *E* ≈ 0.5 MPa, Poisson's ratio *ν* ≈ 0.49. Cotton Fiber modeled as a linear elastic material with a Young’s modulus of 7 GPa and a Poisson’s ratio of 0.3. Electrodes were treated as rigid bodies with structural steel properties to ensure minimal deformation. A refined mesh was employed, especially along material interfaces and contact regions to enhance simulation accuracy. Boundary Conditions include two contact pairs: the one between adjacent fiber bundles and the one between fiber bundles and the electrode surface. Fixed constraint was applied to the bottom electrode, preventing *x*-directional displacement. A controlled vertical displacement was applied at the top fibers to simulate compression. In the electrostatic simulation, the voltages of the two electrode plates were set to 1 and 0 V, respectively. For biomechanical simulations, the OpenSim Gait2392 model was scaled to match the anthropometric dimensions of the volunteer. The mass properties of body segments were proportionally adjusted to ensure the total mass aligned with the subject's measured values. A standardized adult male skeletal model (manufactured by GOM GmbH, Germany) was utilized for analysis. This model, originally reconstructed from CT scans and calibrated, was provided as a SolidWorks® file. A fixed constraint (FC) was applied to the proximal tibia, fully restricting translational and rotational displacements at the tibia-talus interface. Ground reaction forces were converted into equivalent forces and moments at the ankle joint and applied to the contact surface between the tibia and talus. Muscle forces were imposed at the tibial origins and insertions of corresponding musculotendinous units.

## Supplementary Information

Below is the link to the electronic supplementary material.

Supplementary file1 (MP4 2720 kb)

Supplementary file2 (MP4 5933 kb)

Supplementary file3 (MP4 3885 kb)

Supplementary file4 (MP4 4599 kb)

Supplementary file5 (DOCX 25241 kb)
