# Supplementary material for: Skin-Inspired Ultra-Linear Flexible Iontronic Pressure Sensors for Wearable Musculoskeletal Monitoring
Source: Nanomicro Lett. 2025 Sep 1;18:55. doi: 10.1007/s40820-025-01887-x (PMC12401823; doi:10.1007/s40820-025-01887-x)
Supplement: Supplementary file 5 — Supplementary file5 (DOCX 25241 kb) [file 40820_2025_1887_MOESM5_ESM.docx]

Supporting Information for

**Skin-Inspired Ultra-Linear Flexible Iontronic Pressure Sensors for Wearable Musculoskeletal Monitoring**

Pei Li^1,2^, Shipan Lang^1^, Lei Xie^3^, Yong Zhang^1,3^, Xin Gou^1,3^, Chao Zhang^1^, Chenhui Dong^2^ and Chunbao Li^2^, *, Jun Yang^1^, *

^1^ Chongqing Institute of Green and Intelligent Technology, Chinese Academy of Sciences, Chongqing 400714, P. R. China

^2^ Department of Orthopedics, the Chinese PLA General Hospital, Beijing 100039, P. R. China

^3^ Department of Optoelectronic Engineering, Chongqing University, Chongqing 400044, P. R. China

*Corresponding authors. E-mail: [lichunbao301@163.com](mailto:lichunbao301@163.com) (Chunbao Li); [jyang@cigit.ac.cn](mailto:jyang@cigit.ac.cn) (Jun Yang)

**Note S1 Pressure-Induced Variation of Unit-Area Capacitance (UAC)**

First, for the double-layer capacitance, we have,

$C=UAC(\rho)\frac{A}{A_{0}}$ (S1)

Where *UAC* is the maximum value of the capacitance per unit area, which is related to the volumetric ion concentration of the ion gel, *A_0_* is the initial contact area between the ion gel and the electrode, and *A* is the actual contact area between the ion gel and the electrode.

First, according to classical electrochemical theory, the double-layer structure at the interface between the electrode and the ion gel is described, envisioning two layers: the inner layer as the Helmholtz layer with an atomic-scale width, and the outer layer as the diffusion layer, theoretically considered semi-infinite. The distribution of anions and cations within this layer follows the Poisson and Boltzmann laws. *UAC* can be expressed as [S1]:

(S2)

where $\varepsilon$ is the relative permittivity, $\varepsilon_{0}$is the vacuum permittivity, z is the charge number of the ion, $\rho$is the ion concentration, k is the Boltzmann constant, T is the absolute temperature, and is the electrostatic potential of the electrode.

Taking into account the movement of ions under pressure, the local ion concentration will undergo changes, thereby altering *UAC*.

In the previous section, we established the relationship between *UAC* and ion concentration *n*. Under the influence of pressure and voltage, ions undergo movement. The convection of ions in the gel depends on the flow regime of ions, which can be categorized into three modes:

1) Macropore Continuum Flow > 50 nm;

2) Mesopore Transition Flow > 2 nm, < 50 nm;

3) Micropore Molecular Flow < 2 nm.

In the ion gel, there is electrolyte in the large polymer chains, and regardless of the size of free ions, external pressure will cause the electrolyte in the gel to move at the same speed. In this section, we describe the convection of electrolyte within the gel using Darcy's law.

The continuum flow model may be challenging to accurately describe the flow of electrolyte in the gel. For example, the typical pore size of the polymer polyvinyl alcohol used in this study is 1.5-3 nm, a size significantly larger than the ion size in the gel. Even for small ions like potassium, there is a filtration effect. To describe this situation, we established a quasi-continuum model. In the third case, in dense conducting polymers, where the interchain spacing is small enough to cause ion exclusion, a modified form of Darcy's law is used to describe this.

When the size of an ion, along with its solvent outer layer, is significant compared to the pore diameter, the pore and the velocity gradients in the pores hinder the movement of ions. The magnitude of hindrance depends on the shape of the ions and their interactions with the pore surfaces. Given that the ratio of pore size to effective ion radius is less than 10, ion movement is in the transitional regime between molecular and viscous flow. Larger ions interact more strongly with the polymer chains, making them move more easily in the gel compared to smaller ions. The transition flow mode is challenging to model, but in certain cases, a quasi-continuum model can be used to describe it, considering solvent flow within the gel and following Darcy's law. If these ions and their solvation shells are relatively large compared to the size of the pores, then the average movement speed of ions may differ from the mean solvent flow speed, as described by the following equation:

$v_{hindred}=\lambda v_{bulk}$ (S3)

Where λ is the hindrance factor^[2]^. This hindrance may arise due to adhesion between the polymer main chain and ions, as well as differences in ion sizes. When ions are larger than the boundary layer of the pores, their velocity may actually increase relative to the average flow velocity.

We can estimate the force F_s_ exerted on ions lagging behind the solvent flow velocity using Stokes' law. Assuming a no-slip condition at the molecular boundary, Stokes' law is reasonable when the effective size of the solute is two to three times that of the solvated molecules. It is important to note that the physical system studied in this paper is very close to this limit. However, adjustments may be made as a more in-depth understanding of this issue is gained. Therefore, we use Stokes' law as a starting point for understanding ion movement in the gel.

For a cation with a radius r, moving at a speed v_+_ in a medium with viscosity η, the force experienced is:

$F_{s}=6\eta\pi r(v-v_{+})$ (S4)

This force balances the ion resistance caused by gel movement,

$F_{H}=v_{+}/\mu$ (S5)

where μ is the migration rate. There exists an Einstein relationship between migration rate and diffusion coefficient,

$\mu=D_{eff}/kT$ (S6)

where k is the Boltzmann constant, and T is absolute temperature. Assuming the combined effect of the resistance from the solution and polymer chains, taking into account that migration rate and diffusion coefficient are inversely proportional to resistance and following the approach used in solid-state physics to combine the influences of migration rate resulting from multiple mechanisms,

$\frac{1}{D_{+}}=\frac{1}{D_{o}}+\frac{1}{D_{eff}}$ (S7)

eliminating the influence of the solution phase and isolating the resistance effect of the solvent itself, the effective diffusion coefficient is estimated as:

$D_{eff}=[\frac{1}{D_{+}}-\frac{1}{D_{o+}}]^{-1}=\frac{D_{+}D_{o+}}{D_{o+}-D_{+}}$ (S8)

In Eq. (S7), D_o+_ is the diffusion coefficient caused by the solution, and D_+_ is the diffusion coefficient of ions in the hydrogel. According to force balance, i.e., using Stokes force to move ions, and polymer chain resistance hindering ion movement, the velocity of ions is given by:

$v_{+}=\frac{D_{+}}{D_{o+}}v,v_{-}=\frac{D_{-}}{D_{o-}}v$ (S9)

According to Darcy's law,

$v=\frac{k}{\nabla P}$ (S9)

where k is the Boltzmann constant, $\nabla P$ is the pressure gradient. Therefore, the velocity of ion motion can be written as:

$v_{+}=\frac{6\eta\pi r\mu v}{6\eta\pi r\mu+1}=\frac{6\eta\pi r\mu k}{(6\eta\pi r\mu+1)\nabla P}$ (S11)

Assuming ions in the ion gel are initially uniformly distributed, we can simplify the three-dimensional ion gel into a two-dimensional cross-sectional model. Let's consider an infinitesimally thin layer located at distance h1 from the electrode, as illustrated in **Fig. S**1. In unit time, the volume fraction increment in this thin layer determines the ion concentration within it.

The variation in ion concentration in the thin layer is then the difference between the circular areas leaving the thin layer and entering the thin layer. Assuming an equal number of ions in each layer, ions move layer by layer under pressure. It is essential to note that, in practical scenarios, due to the uneven distribution of polymers and pressure, achieving the ideal condition of pressure gradient only along the vertical direction, as assumed in this analysis, is challenging. To demonstrate the theoretical correlation between pressure gradient and ion concentration, we simplified the pressure gradient in the analysis. This simplification can be recalculated using simulation methods to obtain a more accurate ion concentration distribution.

As depicted in **Fig. S**2, according to the formula for the chord length of a circle, we have:

$d=R-v_{+}(h_{1})t$ (S12)

$l=\sqrt{R^{2}-[R-v_{+}(h_{1})t]^{2}}$ (S13)

Therefore,

$\Delta S=S_{2}-S_{1}=\frac{\theta_{2}}{\pi}R-\frac{l_{2}d_{2}}{2}-\frac{\theta_{1}}{\pi}R+\frac{l_{1}d_{1}}{2}$ (S14)

We get,

$\Delta\rho=\frac{2\sqrt{R^{2}-[R-v_{+}(h_{1})t]^{2}}\cdot[R-v_{+}(h_{1})t]}{L\cdot\Delta h}-\frac{2\sqrt{R^{2}-[R-v_{+}(h_{2})t]^{2}}\cdot[R-v_{+}(h_{2})t]}{L\cdot\Delta h}$ (S15)

This equation characterizes the relationship between the pressure gradient and ion concentration.

**Note S2 Pressure-Induced Variation of Contact Area**

We decompose the mechanical model of the sensor into two parts: one is the compressive action between the fiber bundles, leading to a localized change in ion concentration on the surface of the fiber bundles' ion gel. The other part is the variation in a single fiber bundle under compression, causing a change in the contact area between the charged fiber bundle and the electrode, as in **Fig. S**3.

Consider the ion concentration changes caused by the compression between charged fiber bundles. According to the Hertzian contact model, we have,

$m=\lambda\left[P^{2}\left(\frac{r_{1}+r_{2}}{2r_{1}r_{2}}\right)\left(\frac{1-\mu_{1}^{2}}{E_{1}}+\frac{1-\mu_{2}^{2}}{E_{2}}\right)^{2}\right]^{\frac{1}{3}}=\lambda P^{\frac{2}{3}}k_{1}^{\frac{2}{3}}$ (S16)

Where, for simplicity, we abbreviate Eq. (S16) as,

$k_{1}=\left(\frac{1-\mu_{1}^{2}}{E_{1}}+\frac{1-\mu_{2}^{2}}{E_{2}}\right)$ (S17)

where $\mu_{1}$ and E_1_ are the Poisson's ratio and Young's modulus of the iontronic fiber bundle, respectively. The force Q applied to a single fiber bundle is given by,

$Q=\frac{P}{\alpha\left[P\left(\frac{2r_{1}r_{2}}{r_{1}+r_{2}}\right)\left(\frac{1-\mu_{1}^{2}}{E_{1}}+\frac{1-\mu_{2}^{2}}{E_{2}}\right)\right]^{\frac{1}{3}}}=\alpha^{-1}\cdot P^{\frac{2}{3}}\cdot k_{1}^{\frac{1}{2}}$ (S18)

Then, we solve for the contact area between a single fiber bundle and the electrode, as shown in **Fig. S**4.

We get,

$S_{contact}=\left[\frac{4}{\pi}r_{0}\left(\frac{1-\mu_{3}^{2}}{E_{3}}+\frac{1-\mu_{4}^{2}}{E_{4}}\right)Q\right]^{\frac{1}{2}}*l=l\sqrt{\frac{4r_{0}k_{2}}{\pi}}\cdot Q^{\frac{1}{2}}$ (S19)

Substituting Eq. (S18), we can derive,

$S_{contact}=l\sqrt{\frac{4r_{0}k_{2}}{\pi}}\cdot(\alpha^{-1}\cdot P^{\frac{2}{3}}\cdot k_{1}^{\frac{1}{2}})^{\frac{1}{2}}=l\left[\frac{4r_{0}k_{2}\alpha^{-1}}{\pi}\right]^{\frac{1}{2}}\cdot P^{\frac{1}{3}}\cdot k_{1}^{\frac{1}{4}}$ (S20)

For simplicity, let,

$k_{2}=\left(\frac{1-\mu_{3}^{2}}{E_{3}}+\frac{1-\mu_{4}^{2}}{E_{4}}\right)$ (S21)

Therefore, combining with Eq. (S1), we have,

$C=m\cdot\lambda P^{\frac{2}{3}}k_{1}^{\frac{2}{3}}\cdot UAC_{0}\left[\frac{4r_{0}k_{2}\alpha^{-1}}{\pi}\right]^{\frac{1}{2}}\cdot P^{\frac{1}{3}}\cdot k_{1}^{\frac{1}{4}}=\frac{m\lambda k_{1}^{\frac{11}{12}}\cdot UAC_{0}}{A_{0}}\left[\frac{4r_{0}k_{2}\alpha^{-1}}{\pi}\right]^{\frac{1}{2}}P$ (S22)

Therefore, the sensitivity is,

$S=\frac{1}{C_{0}}\frac{\partial C}{\partial P}=\frac{m\lambda\left(\frac{1-\mu_{1}^{2}}{E_{1}}+\frac{1-\mu_{2}^{2}}{E_{2}}\right)^{\frac{11}{12}}\cdot UAC_{0}}{C_{0}A_{0}}\left[\frac{4r_{0}\left(\frac{1-\mu_{3}^{2}}{E_{3}}+\frac{1-\mu_{4}^{2}}{E_{4}}\right)\alpha^{-1}}{\pi}\right]^{\frac{1}{2}}$ (S23)

**Note S3 Young’s Modulus-Induced Variation of Linearity**

The linearity of iontronic pressure sensors is determined by the synergy between mechanical deformation and ionic redistribution under loading. In our architecture, a deformable hydrogel layer (Young’s modulus E₁) is laminated onto a stiff woven substrate (modulus E₂), forming a mechanically heterogeneous composite. The total capacitance output is governed by two coupled mechanisms:

$C(P)\propto A(P,E_{1},E_{2})\cdot UAC(P,E_{1}\text{）}$ (S24)

The contact area A(P) between the sensor and electrode arises from pressure-induced deformation, governed by Hertzian contact theory. We expand the expression for A(P) to incorporate two additional physical effects:

$\lambda_{\mathrm{int}}$: an interfacial conformity coefficient (0<$\lambda_{\mathrm{int}}$≤1), capturing how well the ion gel conforms to the fabric;

$\chi$: a fabric compliance factor (0<$\chi$≤1), reflecting the microstructural compressibility of the textile.

For the composite system, we define an effective modulus:

(S25)

where φ₁ is the volume fraction of the hydrogel. The area scales with pressure as:

$A(P)\propto(P/E_{eff}\cdot\lambda_{\mathrm{int}}\cdot\chi)^{\frac{1}{3}}$ (S26)

Concurrently, the applied pressure compresses the hydrogel matrix, modulating local ionic concentration. Based on modified Poisson–Boltzmann theory and Darcy-Stokes flow, we obtain:

$UAC(P)\propto(\frac{P}{E_{1}})^{\frac{2}{3}}$ (S27)

Combining both, we obtain an overall pressure–capacitance relationship:

his yields an overall pressure-capacitance relationship of:

$C(P)\propto P\cdot E_{eff}^{-\frac{1}{3}}\cdot E_{1}^{-\frac{2}{3}}\cdot\lambda_{\mathrm{int}}^{\frac{1}{3}}\cdot\chi^{\frac{1}{3}}$ (S28)

This derivation demonstrates that linearity is optimized when the fabric provides high modulus, high compressibility (χ), and strong adhesion ($\lambda_{\mathrm{int}}$) to enable synchronized mechanical and ionic responses.

This five-factor model reveals that linearity depends not only on modulus contrast, but also on how effectively mechanical deformation is transferred across the ion gel–fabric interface and distributed across the textile surface.

**Note S4 Data processing**

The data processing workflow is shown in **Fig. S9**. Utilizing foot pressure data acquired during human motion to extract the stance phase and swing phase within a gait cycle. In the preliminary data analysis, significant jitter was observed in the signals. Therefore, the signals are first smoothed using triple mean filtering and median filtering, which can preserve the original characteristics of the signals and facilitate subsequent feature extraction.

Combining the description of gait phase timing and the analysis of force variations at different positions on the foot during the gait cycle, the peak and valley positions of the signals obtained from the toe and heel sensors can be used to calculate the stance phase and swing phase at different walking speeds. Different strategies are applied to extract the peak and valley positions for the signals from the toe and heel sensors. For the toe sensor signal, a neighboring value comparison method is used, followed by amplitude-based outlier removal using adjacent peak and valley values. The same approach is applied to the heel signal to obtain the positions of its peaks and valleys. Then, by cumulatively summing the times of individual gait cycles obtained from the toe sensor signal, the peak and valley positions of the heel signal are obtained.

Two adjacent peak points in the heel signal can determine a gait cycle. The stance phase of a single gait cycle can be determined by calculating the time ratio of the distance between the heel signal peak and the subsequent toe signal peak and valley within a gait cycle. Similarly, the swing phase can be determined by calculating the time ratio of the distance between the toe signal peak and valley and the next heel signal peak within a gait cycle.

**Note S5 Algorithms**

1. **SVM Model for Gait Classification**

**Input:**

Time-series plantar pressure data from the 8 sensing channels were segmented using a sliding window approach, with a window size of 60 time steps (sampling rate: 100 Hz). Each sample input is therefore a matrix of shape.

**Feature Extraction:**

A set of representative machine learning features were extracted from each window, including:

Time-domain features: mean, variance, maximum, and skewness of each channel.

Frequency-domain features: power spectral density and spectral entropy.

Spatial features: center of pressure (CoP) trajectory and distribution.

**Output Classes:**

The model was trained to classify gait conditions such as different speeds and slopes with each class encoded using numerical labels.

**Loss Function:**

During training, the core optimization objective of the Support Vector Machine (SVM) is to minimize a loss function based on hinge loss. This loss function consists of two main components.

A regularization term representing the squared L2 norm of the weight vector, which controls the model complexity, as shown below:

(S29)

An empirical risk term that penalizes classification errors or margin violations, formulated as:

(S30)

The final loss function is expressed as follows,

(S31)

Where $\mathbf{w}\cdot\mathbf{x}_{i}+b$denotes the decision function for sample $\mathbf{x}_{i}$, and $y_{i}\in-1,+1$ represents the binary classification label (in multi-class scenarios, the problem is transformed into binary form). The regularization parameter The regularization parameter 𝐶 is selected via grid search within the range {0.1, 1, 10}.

1. **TCNformer Model for GRF Prediction**

**Input:**

The same pressure data matrices were used as input to the model. The label for each sample is the vertical ground reaction force (vGRF) value at the final time step of the window, making the output shape per sample.

**Model Architecture:**

The proposed TCNformer comprises four modules:

Preprocessing Module: Filtering, downsampling, and windowing using a `DataLoader` to feed batches into the model.

TCN Module: A 1D convolutional temporal network to capture short-term dependencies in the time series.

Transformer Encoder Module: Stacked encoder blocks with multi-head self-attention for modeling long-range temporal patterns.

Regression Head: A linear projection of the encoded features to predict vGRF or tibial load values.

**Loss Function:**

We propose a Weighted MSE Loss to improve the model’s sensitivity to peak forces. Given that physiological force peaks are crucial in biomechanical monitoring, we assign higher weights to time steps around vGRF peaks.

The weighted MSE loss is defined as:

$\text{Weight-MSELoss}=\frac{1}{T}\sum_{t=1}^{T} w_{t}\cdot\left(y_{t}-\hat{y}_{t}\right)^{2}$ (S32)

**Peak Detection:**

To define peak regions robustly:

For vGRF, peak duration was set to ≥12 time steps (\~120 ms);

For tibial load, due to sharper transitions, the threshold was ≥8 time steps.

This peak-aware loss encourages the model to prioritize prediction accuracy at key biomechanical events without ignoring the global signal trend.

These additions ensure that our SVM and TCNformer models are fully reproducible and transparent for future studies. We thank the reviewer for encouraging us to clarify this aspect, which strengthens the methodological rigor of our work.

1. **Pseudo-Label Generation and Uncertainty Estimation**

To estimate the confidence of model predictions on unlabeled outdoor data, we employed the Monte Carlo Dropout (MC-Dropout) technique. Unlike standard inference, where Dropout is disabled, MC-Dropout retains stochasticity during inference, enabling multiple forward passes on the same input to construct a distribution over predictions. For each unlabeled input sample, we performed T = 10 stochastic forward passes, yielding predictions $\hat{y}_{i}$.The pseudo-label $\bar{y}$ and the corresponding standard deviation$\sigma^{2}$ were calculated as:

$\bar{y}=\frac{1}{T}\sum_{i=1}^{T} \hat{y}_{i},\sigma^{2}=\frac{1}{T}\sum_{i=1}^{T} (\hat{y}_{i}-\bar{y})^{2}$ (S33)

To evaluate the prediction uncertainty, we computed the coefficient of variation (CV)：

$\text{CV}=\frac{\sigma}{|\bar{y}|}\times100\%$ (S34)

The confidence score conf∈[0,1] was derived from CV using a piecewise linear mapping:

$\text{conf}=\text{clip}\left(1-\frac{\text{CV}-\text{CV}_{\text{low}}}{\text{CV}_{\text{high}}-\text{CV}_{\text{low}}}, 0, 1\right)$ (S35)

where we set: $\text{CV}_{\text{low}}$=5%,$\text{CV}_{\text{high}}$=15%. This strategy ensured that only high-confidence pseudo-labels (conf > 0.5) were retained for further training. Out of 5,286 total outdoor samples, 1,045 high-confidence samples (19.77%) were selected for semi-supervised fine-tuning. As illustrated in **Fig.** S, one such sample (ID: 63) shows a tight distribution of predictions around its mean (33,419.2), reflecting high certainty and thus qualifying for pseudo-labeling.


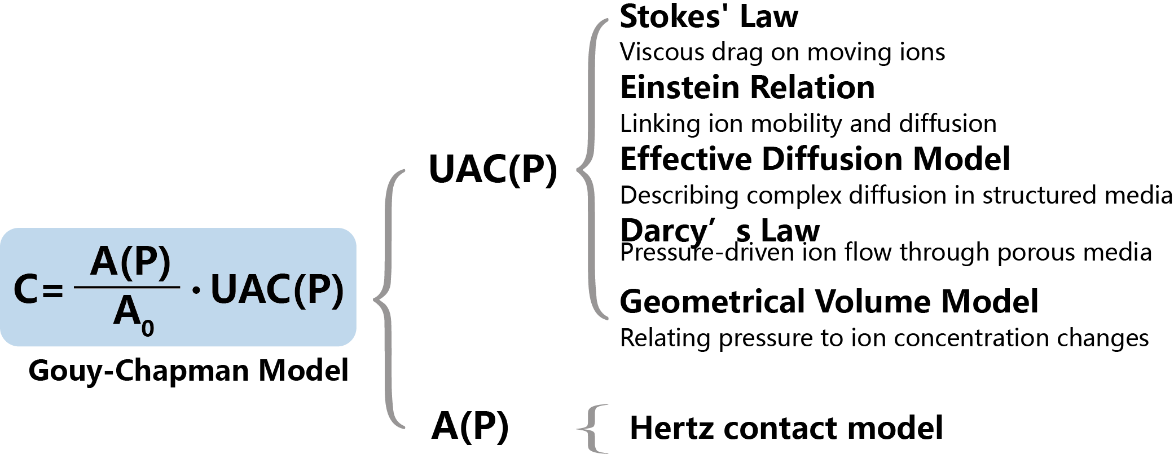


**Fig. S**1 Derivation flowchart of the dual-mechanism model

**Fig. S**2 Cross-section of the ion gel thin layer

**Fig. S**3 Volume fraction change of 2 ions in the thin layer

**Fig. S**4 Interwoven iontronic fiber bundles

**Fig. S**5 Variation in the contact area of a single fiber bundle


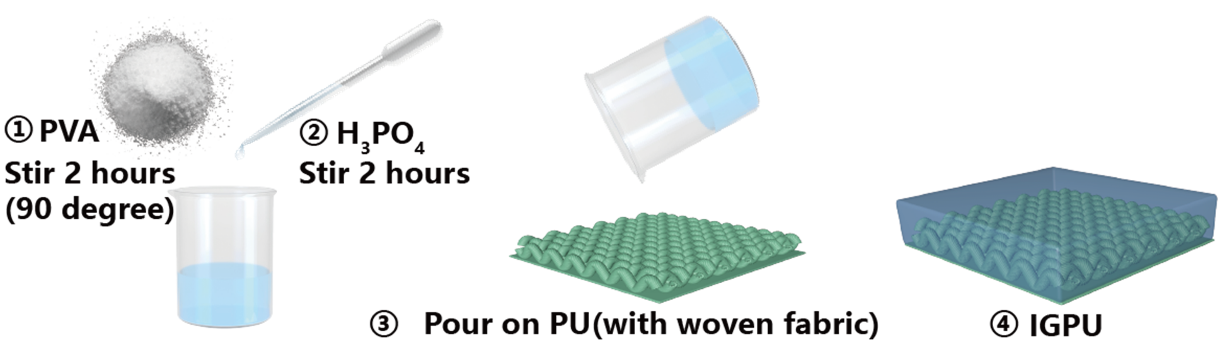


**Fig. S**6 Production processes of iontronic fabric

**Fig. S**7 Evolution of contact area between the sensor structure and the electrodes with increasing displacement


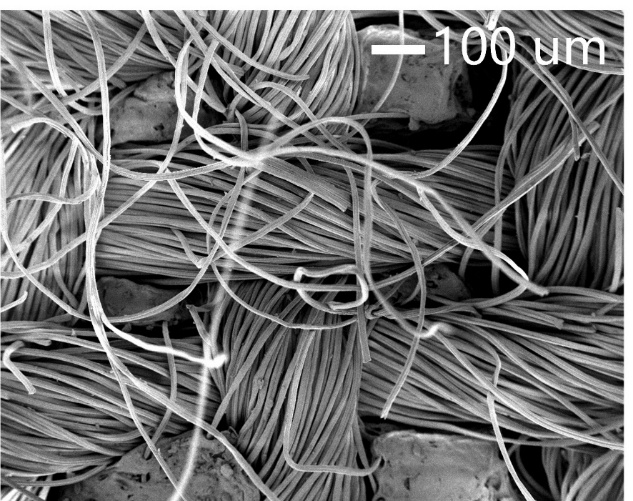


**Fig. S**8 SEM image of the structure of synthetic leather, showing the interweaving of warp and weft threads in the leather, as well as the bundling of yarns in the warp and weft


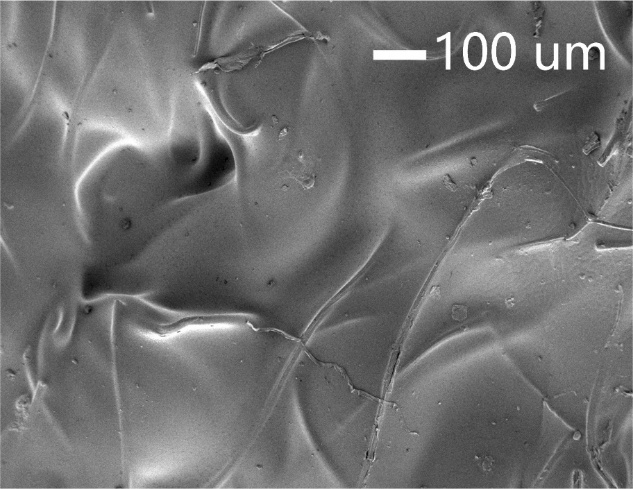


**Fig. S**9 Surface image of Iontronic fabric after casting with ion gel, demonstrating complete immersion of the fiber surface in the ion gel


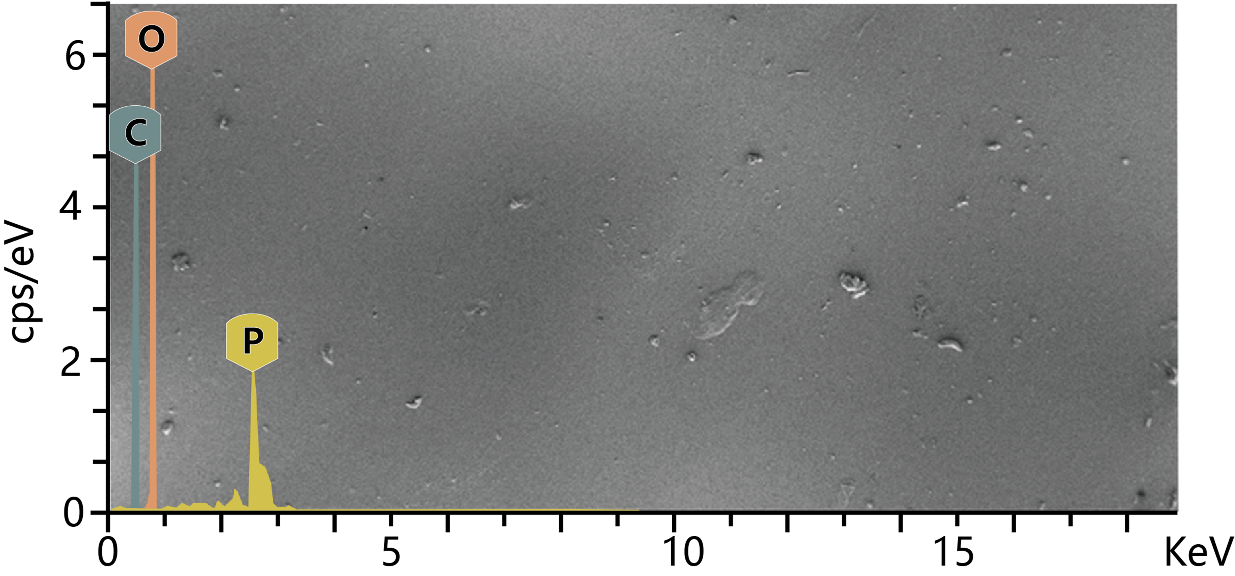


**Fig. S**10 Energy spectrum of the ion gel part of the Iontronic fabric.


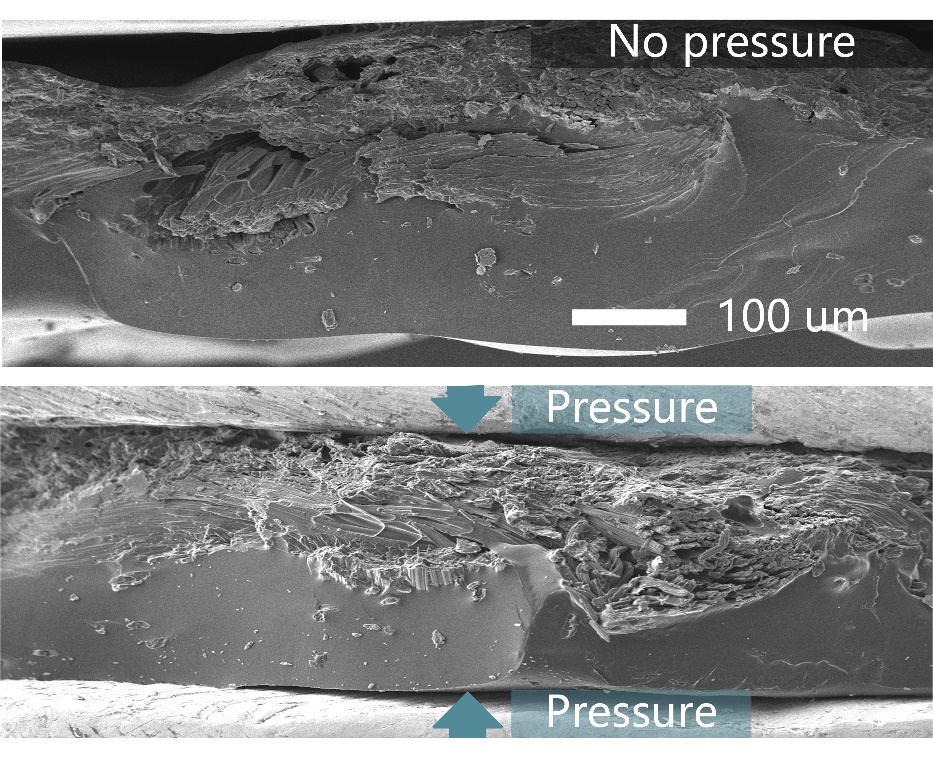


**Fig. S**11 Structural changes of iontronic Fabric before and after compression. After being compressed, the ionic gel layer beneath the fabric is flattened, leading to a change in its contact surface area with the electrode. Additionally, the woven layer of the iontronic Fabric experiences reduced gaps and becomes tighter upon compression. It can be observed that at this point, the gel layer also deforms after being compressed, and at the intersections of the fabric weave, the gel is at its thinnest, making it more susceptible to deformation, resulting in changes to the bulk ionic density.

**Fig. S**12 Sensor’s performance of PVDF ion gel leather with different knitting methods. (**a**) PG1 with PVDF ion gel; (**b**) The performance of PG1 iontronic sensor, R^2^=0.9993, S= 26.38 kPa^-1^; (**c**) PG2 with PVDF ion gel; (**d**) The performance of PG2 iontronic sensor, R^2^=0.9988, S= 5.37 kPa^-1^

**Fig. S**13 Sensor’s performance of PVDF ion gel fabric with different knitting methods. (**a**) B1 with PVDF ion gel; (**b**) The performance of B1 iontronic sensor, R^2^=0.9963, S= 8.3 kPa^-1^; (**c**) B2 with PVDF ion gel; (**d**) The performance of B2 iontronic sensor, R^2^=0.9988, S= 14.38 kPa^-1^

**Fig. S**14 Sensor’s performance of PVDF ion gel fabric with different knitting materials. (**a**) Glass fiber fabric;(**b**) PVDF ion gel on glass fabric; (**c**) The performance of glass fiber iontronic sensor, R^2^=0.9715, S= 30.37 kPa^-1^; (**d**) Nylon fiber fabric; (**e)** PVDF ion gel on nylon fabric; (**f**) The performance of nylon iontronic sensor, R^2^=0.9787, S= 0.58 kPa^-1^


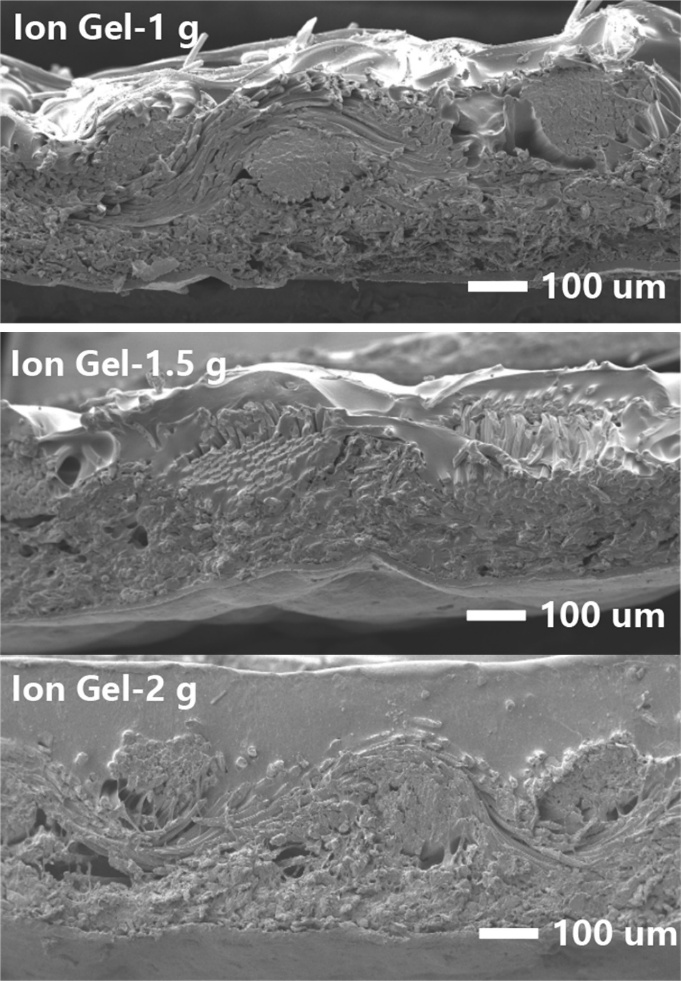


**Fig. S**15 Cross-sectional schematic diagram of iontronic fabric with different ion gel contents. The ion gel content is relatively low on the surface of the 1g iontronic fabric, resulting in a more enriched surface structure. The ion gel thickness is greater in the 1.5g iontronic fabric compared to the 1g fabric. In the 2g iontronic fabric, the ion gel content is higher, resulting in the thickest gel layer, although it does not exceed the thickness of the fiber.


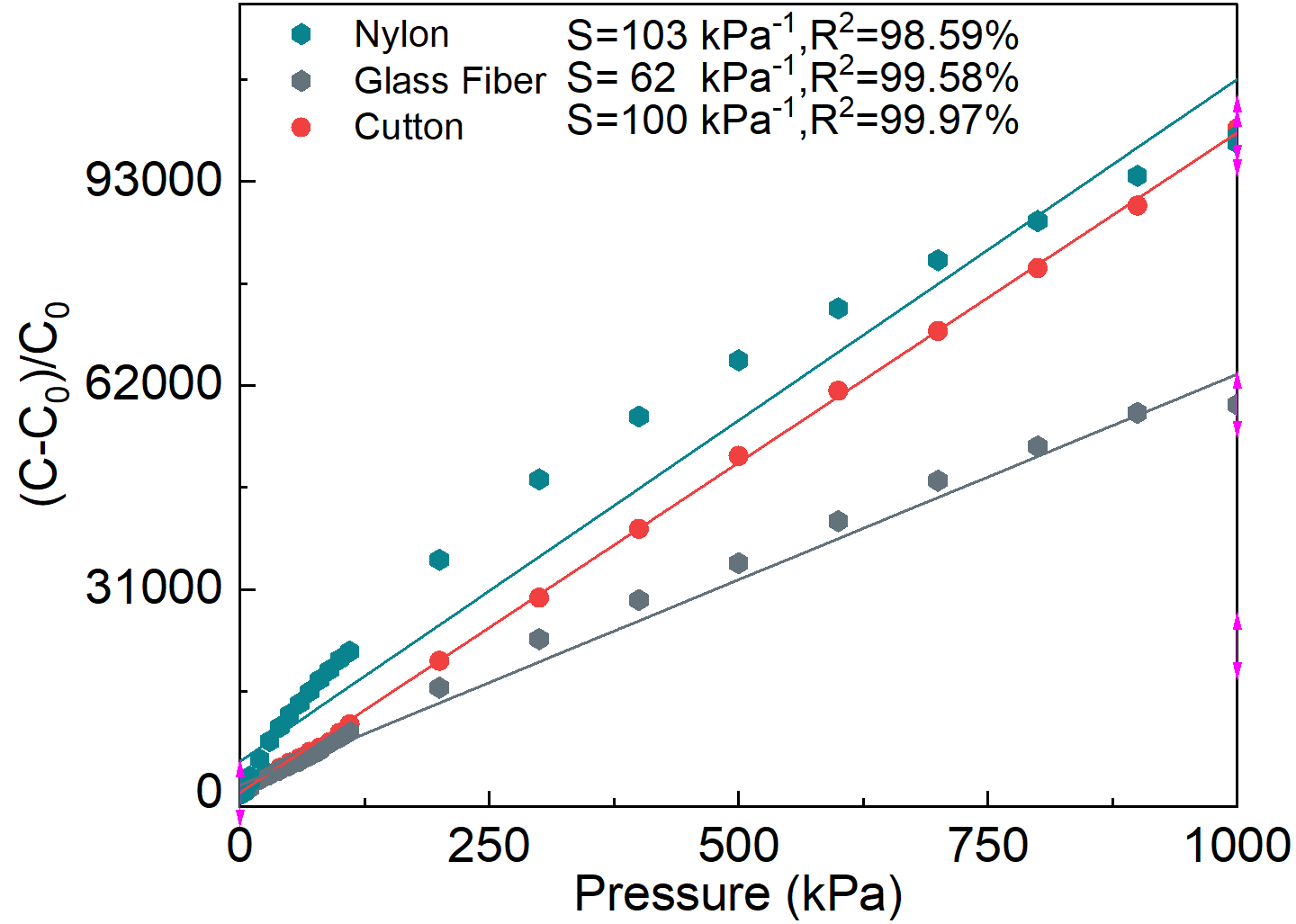


**Fig. S**16 Different iontronic fabrics with the same ion gel

**Fig. S**17 The ionic fabric displays varied sensing performance with different thickness


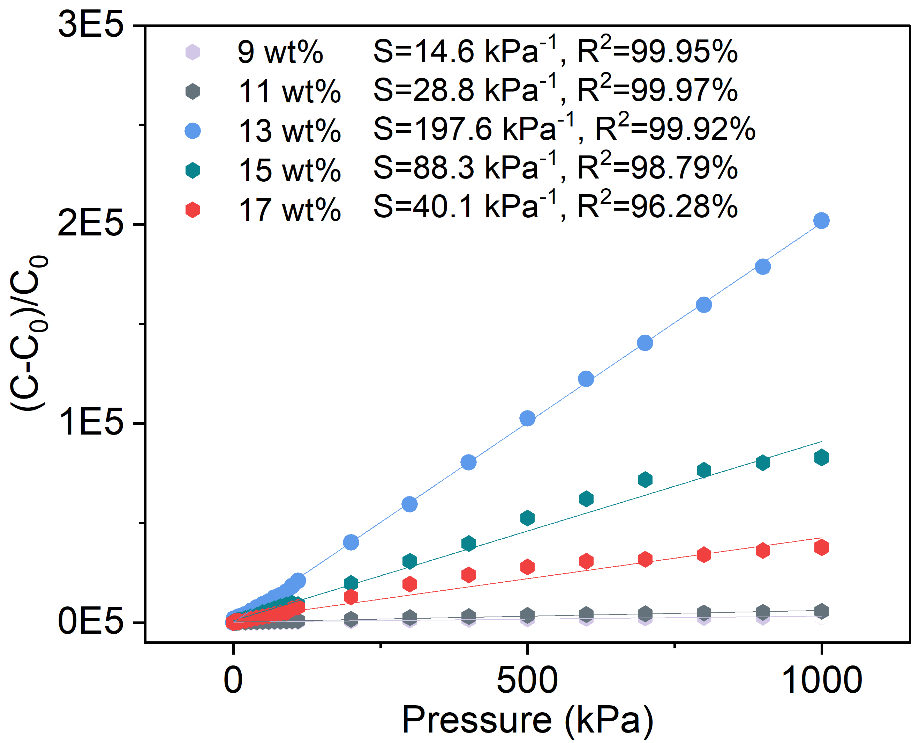


**Fig. S**18 FIPS performance of different H_3_PO_4_ content


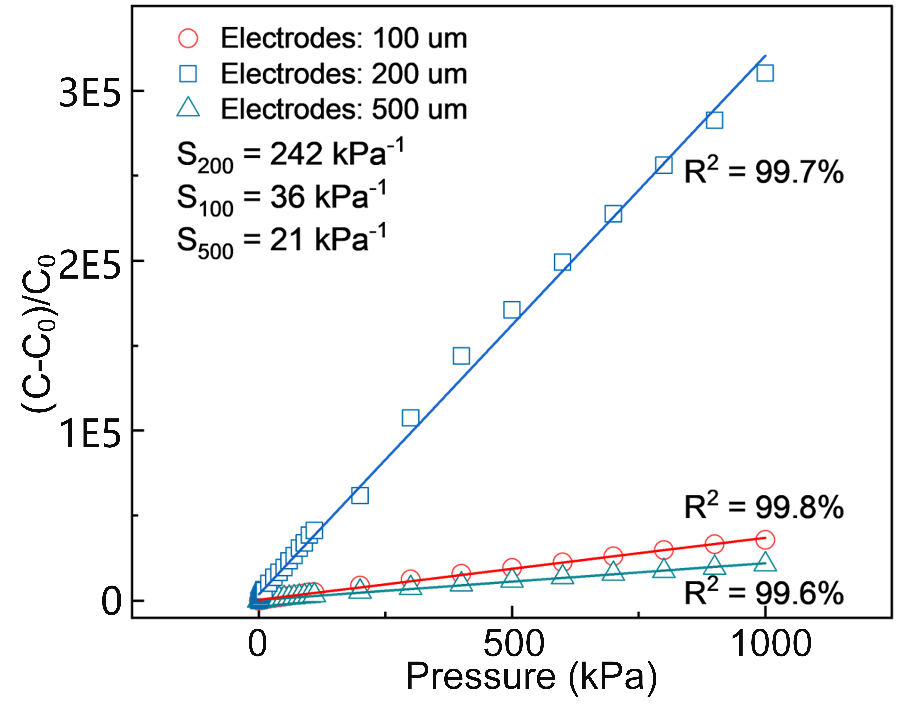


**Fig. S**19 The ionic fabric displays varied sensing performance under interdigital electrodes with different spacings. Devices with an electrode spacing of 100 μm have a sensitivity of 36 kPa^-1^. Those with a spacing of 200 μm exhibit a sensitivity of 21 kPa^-1^, and those with a spacing of 500 μm show a sensitivity of 242 kPa^-1^. Among these, the device with an electrode spacing of 200 μm demonstrates the best performance


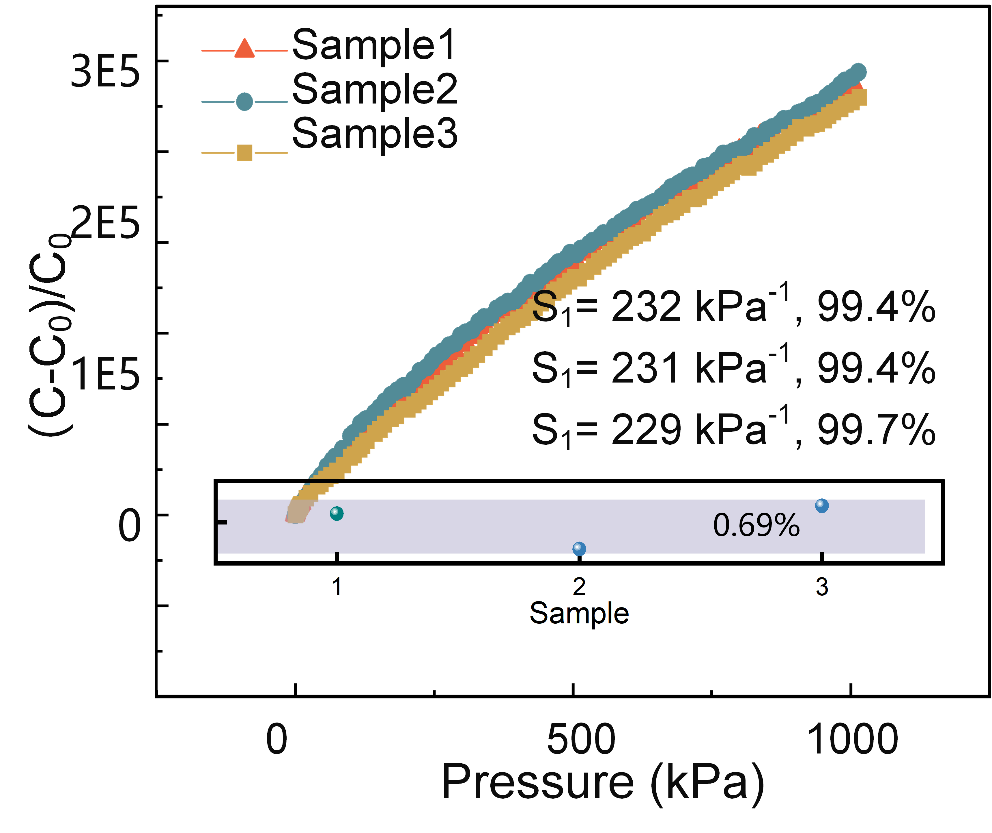


**Fig. S**20 To validate the consistency of sensor fabrication, three FIPS were simultaneously produced in the experiment and their sensitivity curves were measured consecutively. The consistency across the three sensitivity curves was exceptional, with sensitivities of 229 kPa-1, 231 kPa-1, and 232 kPa-1, respectively, showing a difference of less than 0.69%


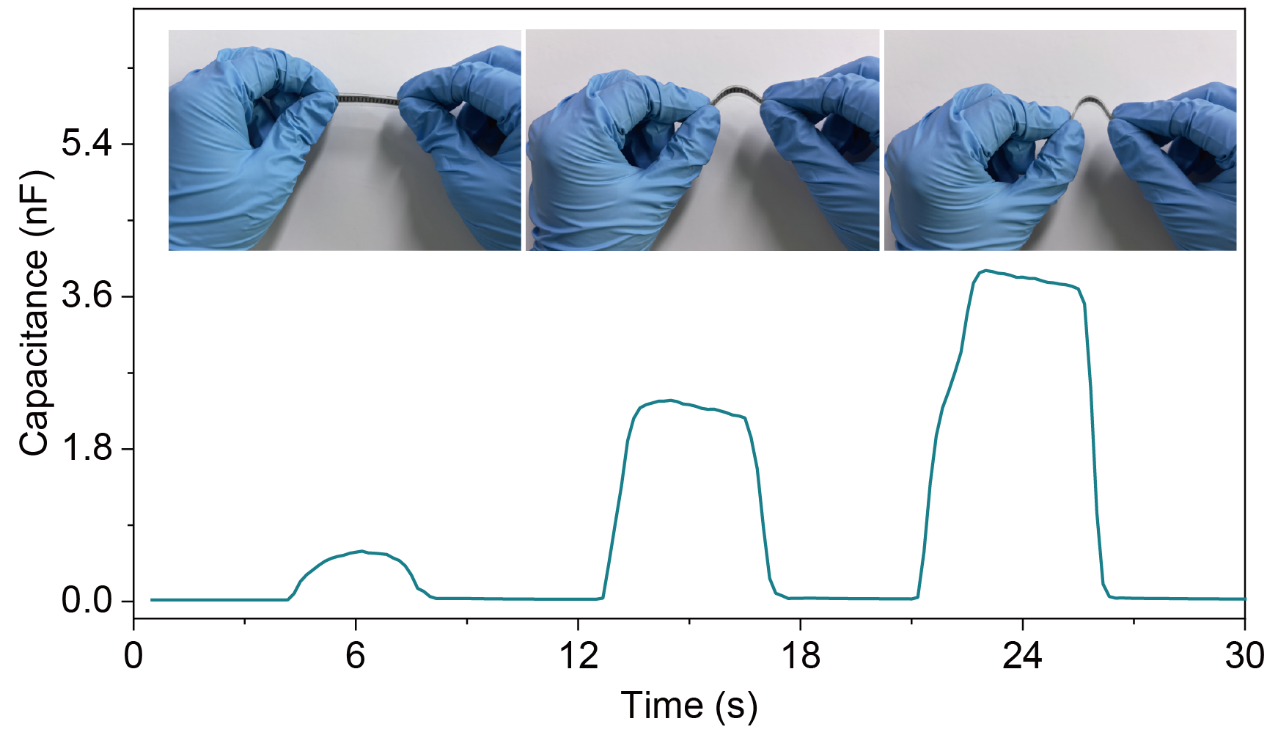


**Fig. S**21 Sensor’s output during bending


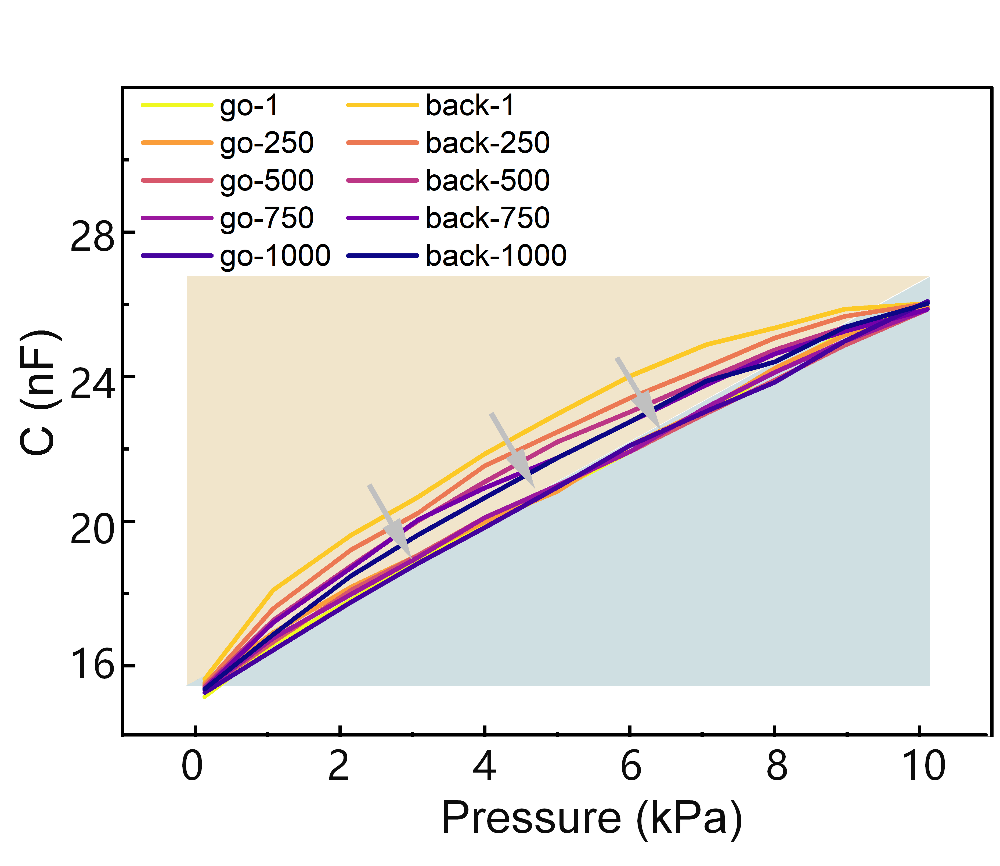


**Fig. S**22 Changes in the hysteresis characteristics of the sensor under 1000 compression cycles. The sensor's loading curve increases linearly with the increase in applied force. During the unloading process, there's a noticeable hysteresis in the sensor's response. This hysteresis diminishes as the number of compression cycles increases


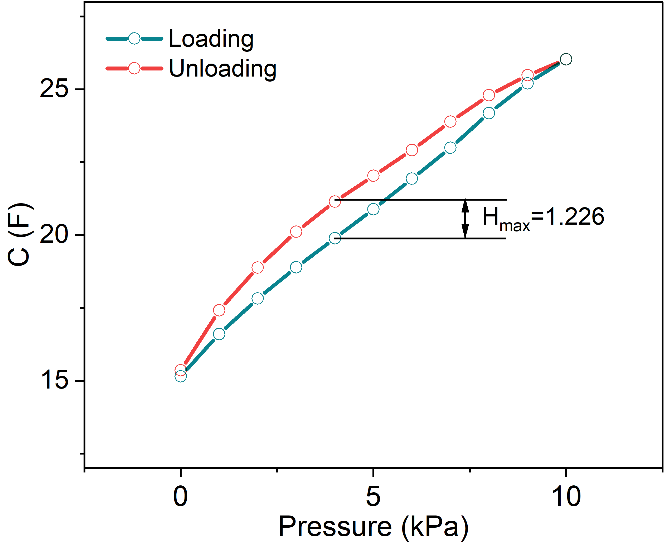


**Fig. S**23 Hysteresis value calculated


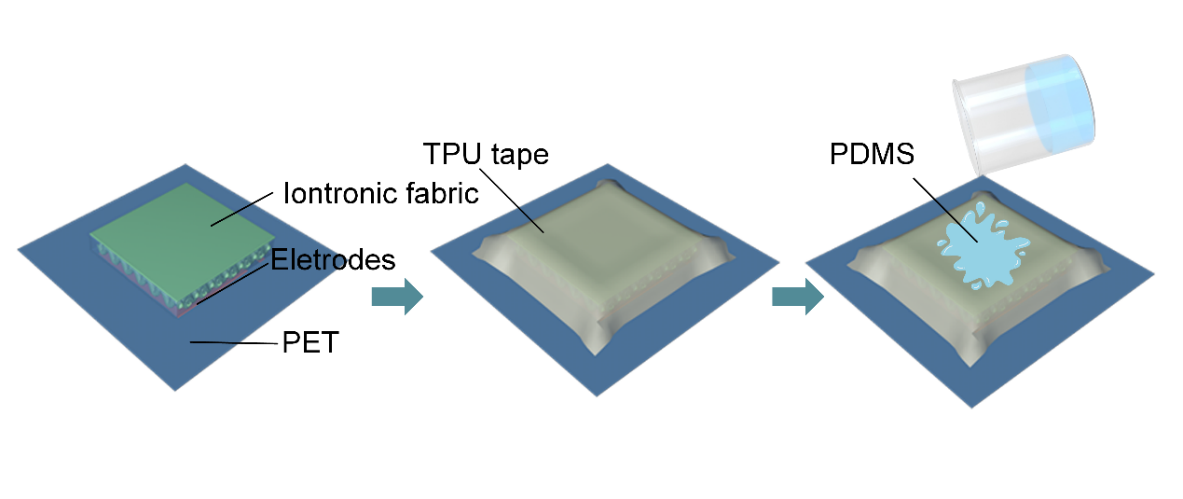


**Fig. S**24 Encapsulate the ionized sensor with TPU tape and PDMS


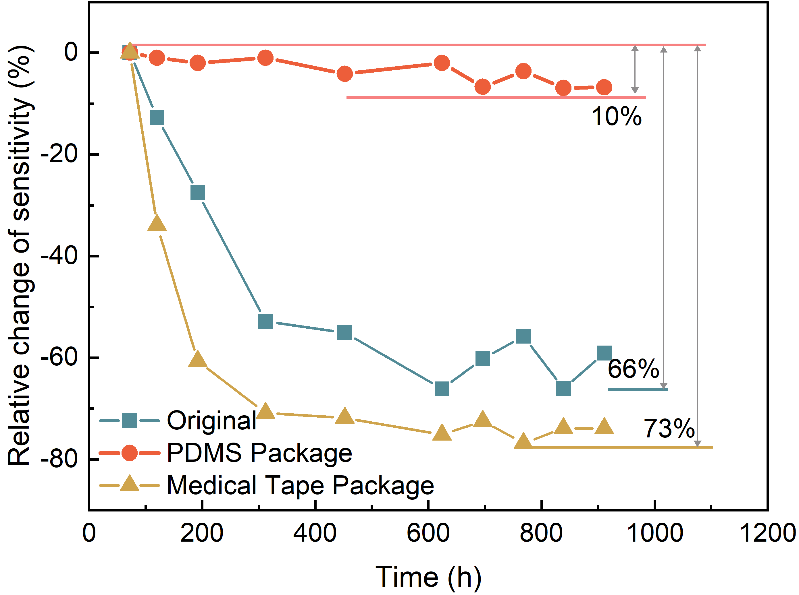


**Fig.** S25 Long term performance of sensors with different packaging


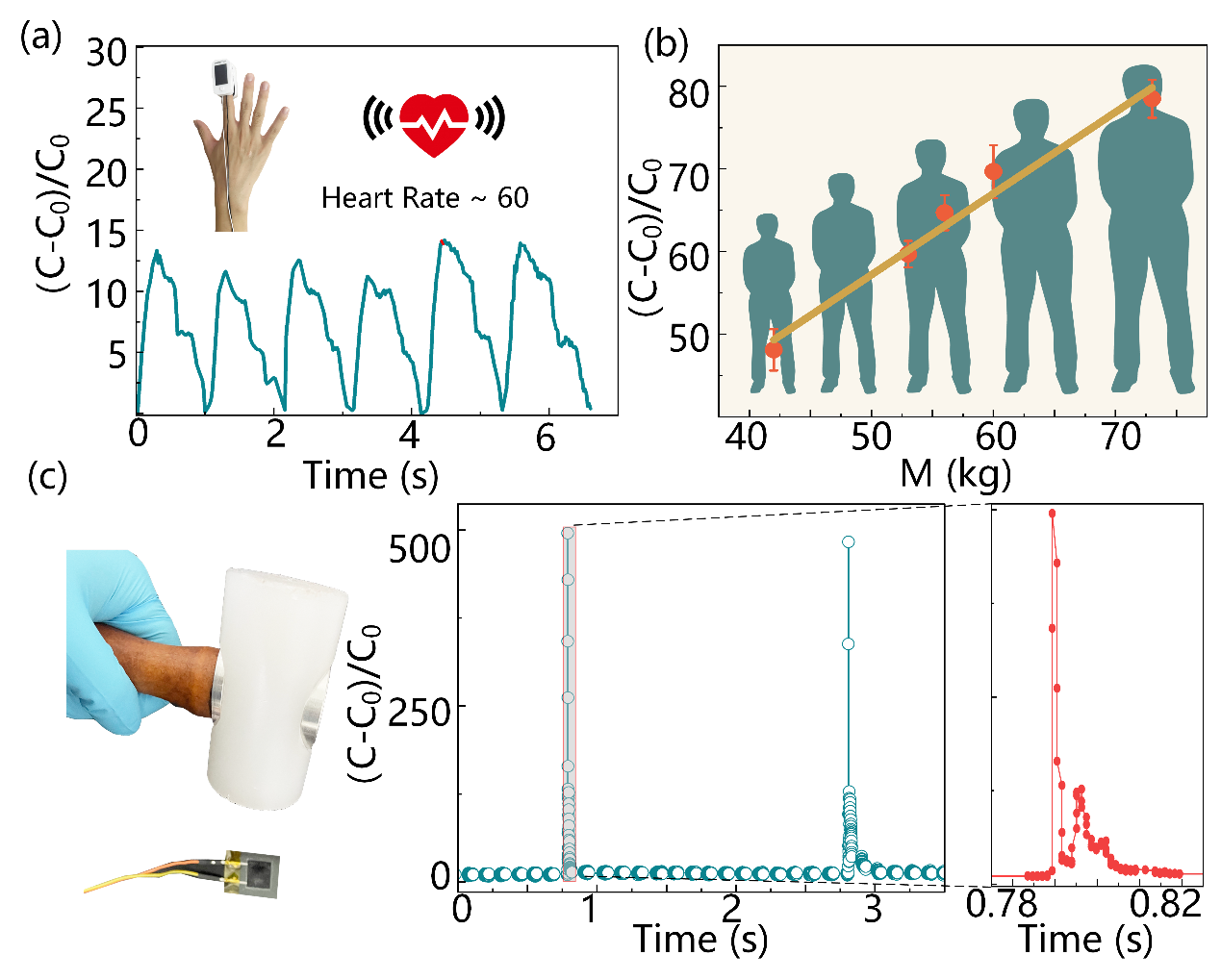


**Fig. S**26 (**a**) Pulse monitoring using the FIPS. (**b**) Body weight measurement demonstrating the excellent linearity of the FIPS. (**c**) High-pressure performance test: the FIPS was placed on a tabletop and struck twice with a nylon hammer. The inset highlights the sensor’s rapid signal response and buffering behavior during the brief impact events


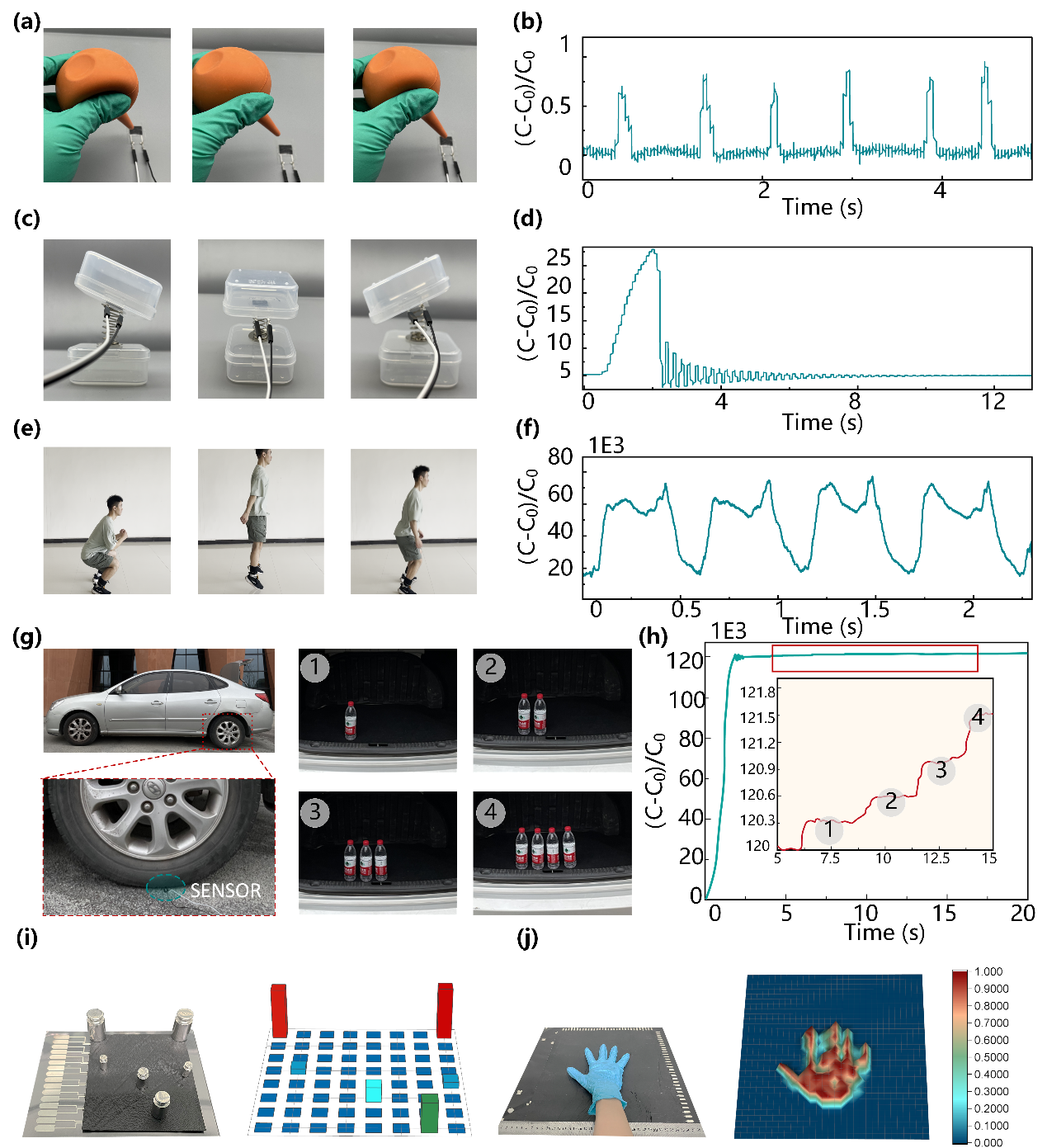


**Fig. S**27 The application of FIPS. (**a**) FIPS beneath an air blower. (**b**) FIPS signal change during squeezing the air blower. (**c**) Putting FIPS on top of a spring. (d) FIPS monitoring the dissipation process of elastic potential energy. (**e**) FIPS are placed on the sole of the foot when volunteers take a jump. (**f**) FIPS collects signals during the process of stepping on the ground and landing buffering. (**g**) FIPS is placed at the bottom of the tire and 500 g of water is sequentially placed in the trunk. (**h**) The FIPS can discern subtle pressure changes even under substantial pressure, and exhibit a high resolution of 0.03%. **(i**) 8×8 FIPS array. (**j**) 32×32 FIPS array

**Fig. S**28 Composition of the sensor system, including a pressure insole made up of FIPS, a detection circuit capable of monitoring capacitive changes (containing a Bluetooth module), and a battery module


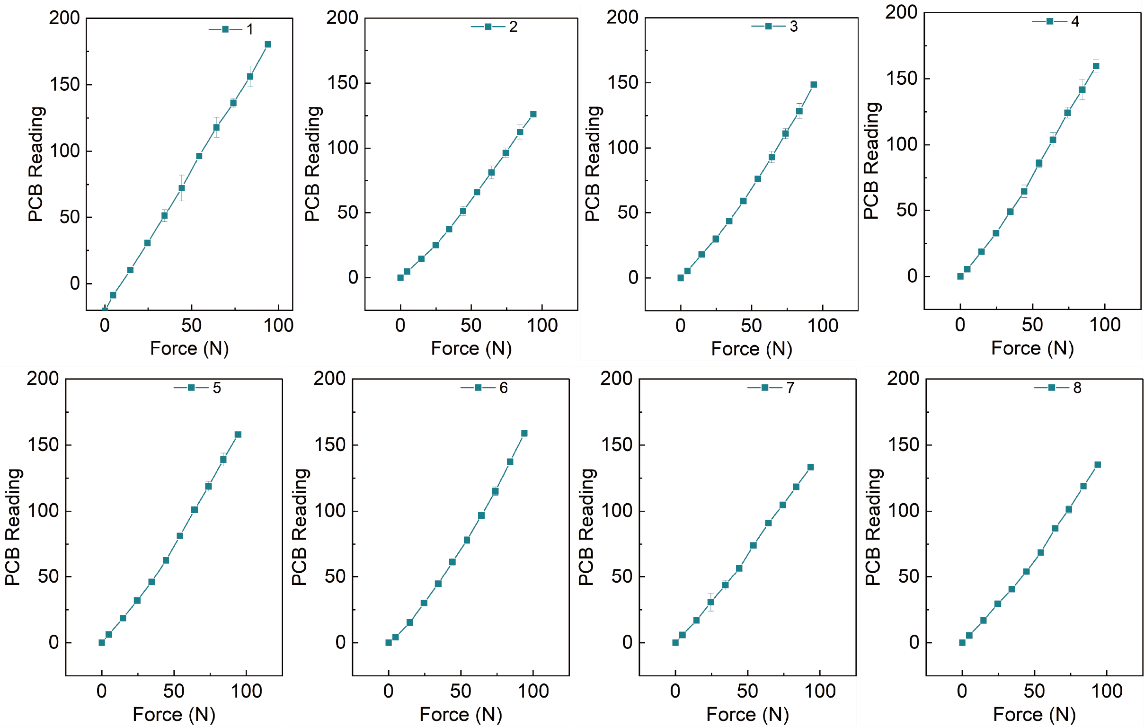


**Fig. S**29 The Linear Calibration of iontronic insole

**Fig. S**30 The application of FIPS in gait monitoring. (**a**) A photograph of a volunteer wearing the FIPS insole sensor. (**b**) The signal acquisition and processing process of FIPS insoles. (**c**) A schematic illustration of the gait cycle analysis


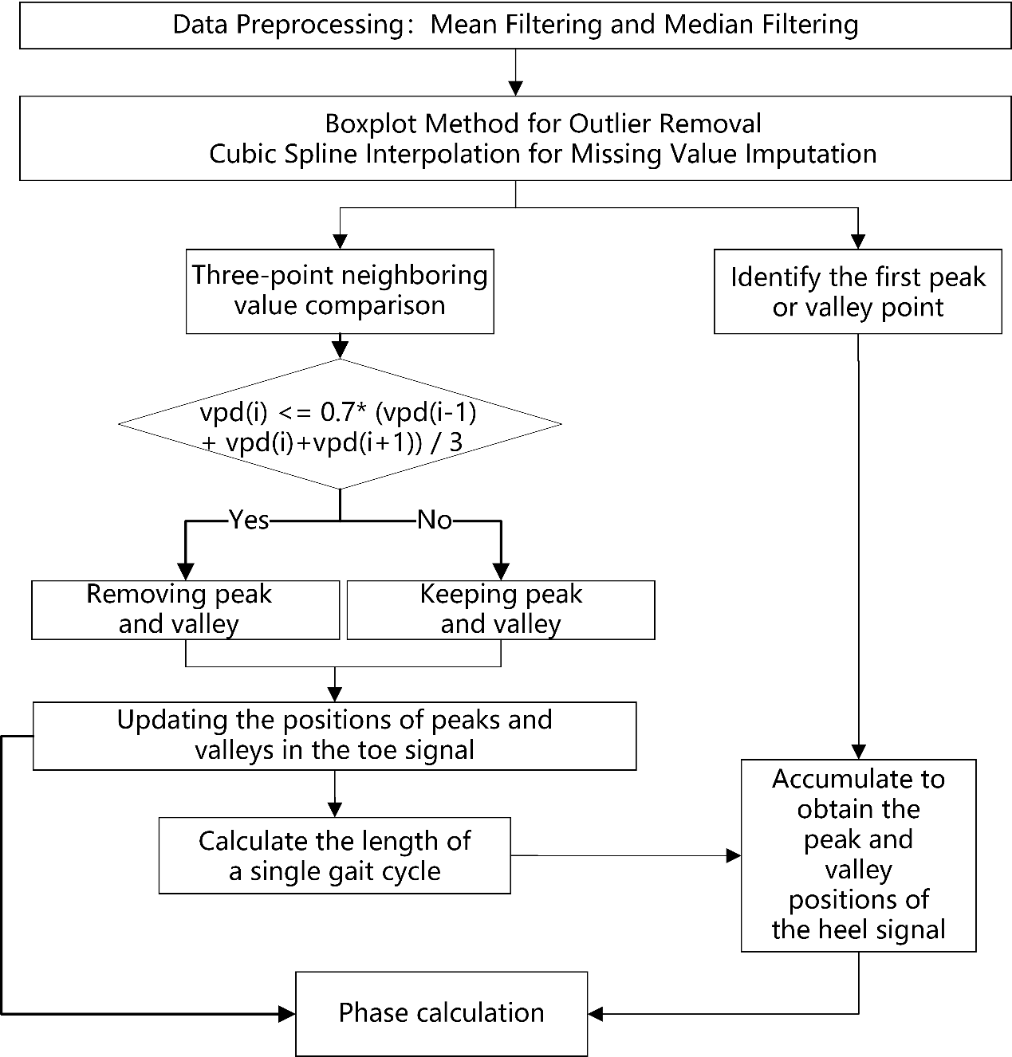


**Fig. S**31 Data Processing Flowchart for Multi-channel Gait Information


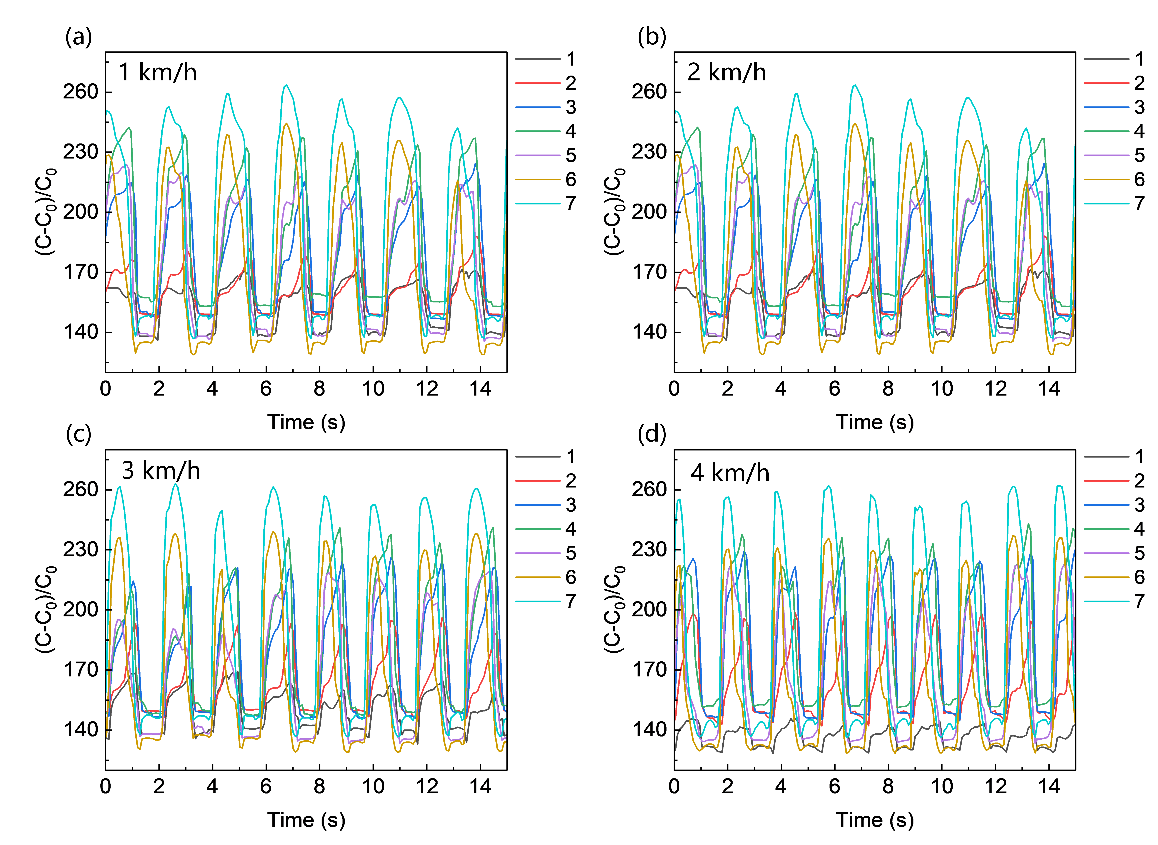


**Fig. S**32 Signal changes of the 7-channel sensor at different walking speeds: 1 km/h, 2 km/h, 3 km/h, and 4 km/h


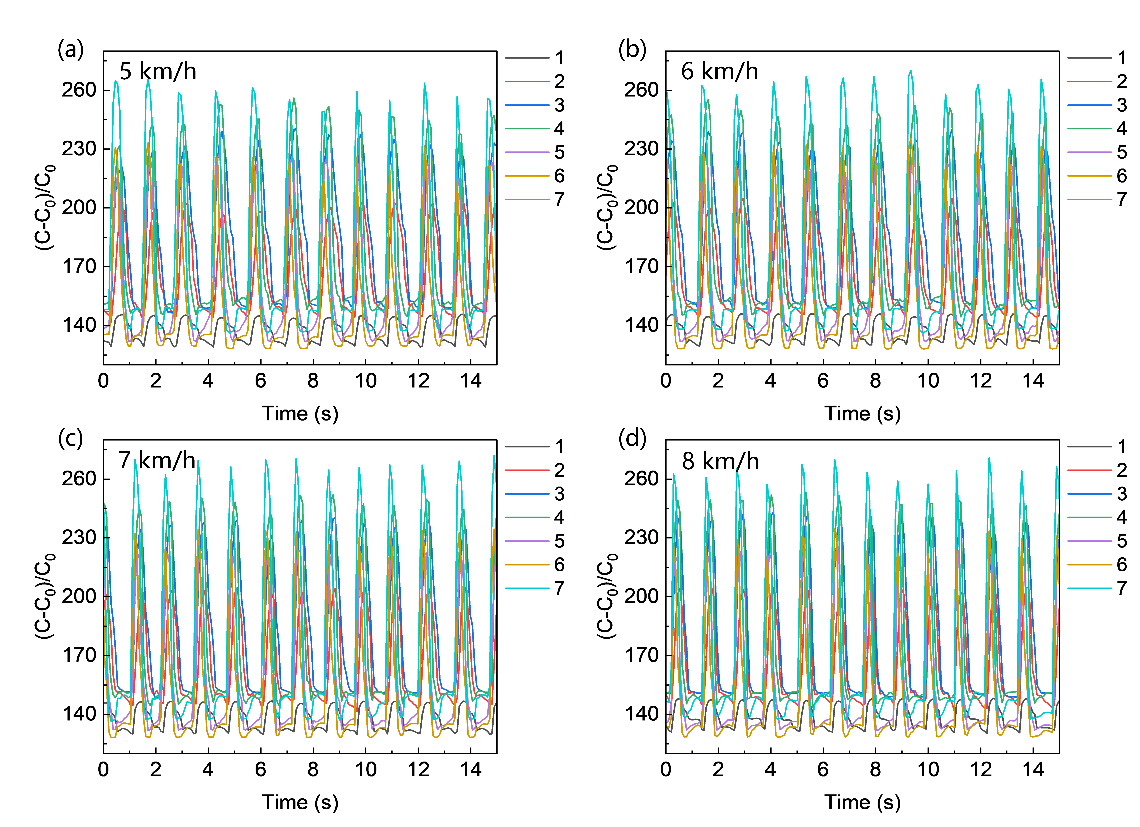


**Fig. S**33 Signal changes of the 7-channel sensor at different walking speeds: 5 km/h, 6 km/h, 7 km/h, and 8 km/h

**Fig. S**34 The working flow of Opensim simulation

**Fig. S**35 The simulation of tibial stress at different times during gait cycle

**Fig. S**36 Von Mises stress at different times on the distal one-third of the tibia


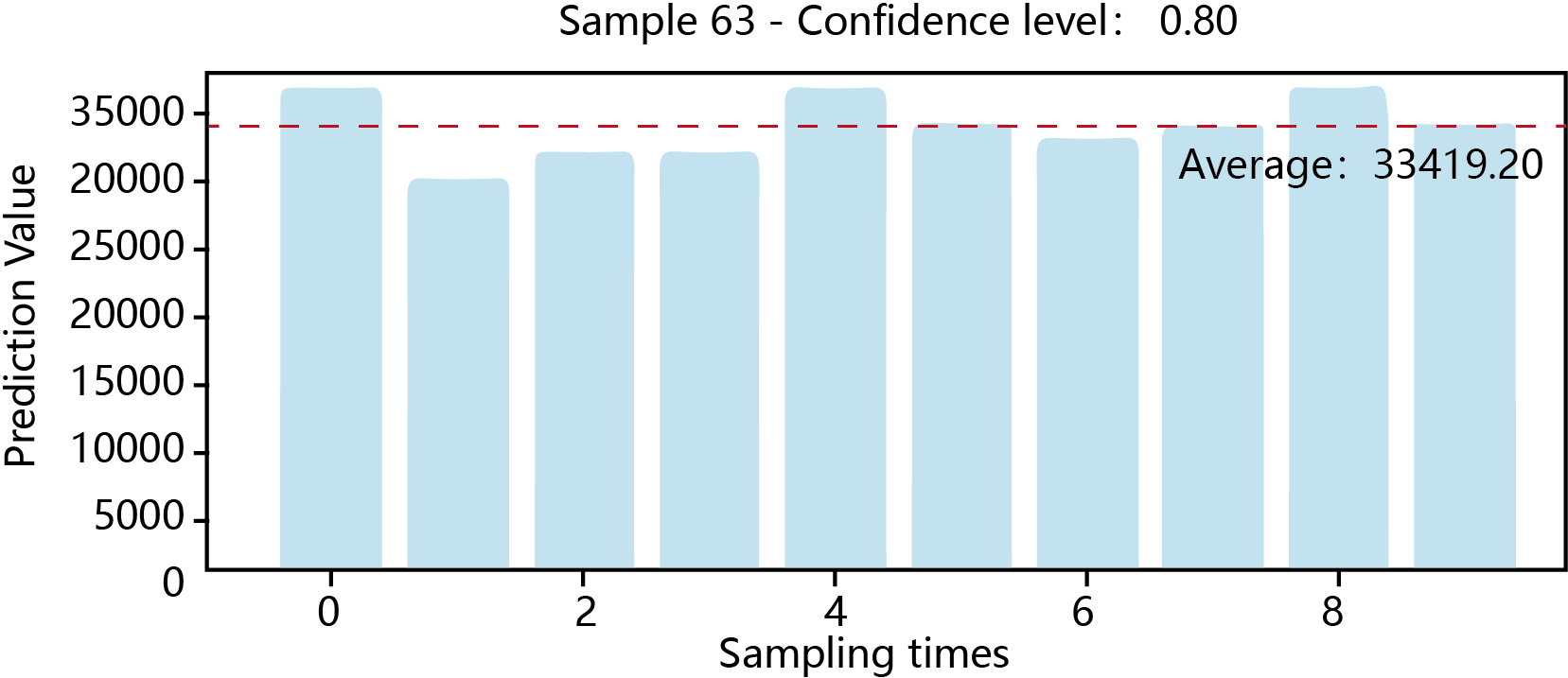


**Fig. S**37 Example of selected samples from outside the laboratory

**Table S1** Parameters of different materials

| Materials | E2 | $\lambda_{\mathrm{int}}$ | $\chi$ |
| --- | --- | --- | --- |
| Cutton | 10 | 0.9 | 0.9 |
| Nylon | 2.5 | 0.6 | 0.6 |
| Glass Fiber | 70 | 0.4 | 0.3 |

**Table S2** Comparison of sensing performance of linear sensors in recent years

| Dielectric  material | Electrode  material | Operating pressure range[kPa] | Maximum sensitivity[kPa^-1^] | | Linearity  R^2^ | Refs. |
| --- | --- | --- | --- | --- | --- | --- |
| CNT/PDMS | Silver‑coated fabric | 800 | 6.4 | - | | [S3] |
| Intronic film | Au/PDMS | 12–176 | 33.16 | 0.999 | | [S4] |
| Iontronic film | Ag/PI with rough surface | 50-200 | 2383 | 0.991 | | [S5] |
| PDMS/ CCP | Cu | 10 | 1.1 | - | | [S6] |
| Iontronic film | Au/PI film | 2063 | 9.17 | - | | [S7] |
| Iontronic film | Au/PDMS | 485 | 49.1 | 0.995 | | [S8] |
| Iontronic film | PI/Gold | 1000 | 12.43 | 0.995 | | [S9] |
| Graphene foams | Silver paste/PDMS | 35 | 134.75 | - | | [S10] |
| Iontronic Fabric | Ag on PET | 1000 | 232 | 0.997 | | This work |

**Table S3** Differences in MAE, RMSE, and R2 of different insoles

| Insole | MAE | RMSE | R2 |
| --- | --- | --- | --- |
| Non-Linear | 38.98 | 67.87 | 0.970 |
| Linear | 10.94 | 12.59 | 0.998 |

**Supplementary References**

1. J. Wu, Understanding the electric double-layer structure, capacitance, and charging dynamics. Chem. Rev. **122**(12), 10821–10859 (2022). <https://doi.org/10.1021/acs.chemrev.2c00097>
2. A.J. Bard, L.R. Faulkner, H.S. White, Electrochemical Methods: Fundamentals and Applications. Hoboken: John Wiley & Sons, (2022).
3. H. Xu, L. Gao, Y. Wang, K. Cao, X. Hu et al., Flexible waterproof piezoresistive pressure sensors with wide linear working range based on conductive fabrics. Nanomicro Lett. **12**(1), 159 (2020). <https://doi.org/10.1007/s40820-020-00498-y>
4. P. Lu, L. Wang, P. Zhu, J. Huang, Y. Wang et al., Iontronic pressure sensor with high sensitivity and linear response over a wide pressure range based on soft micropillared electrodes. Sci. Bull. **66**(11), 1091–1100 (2021). <https://doi.org/10.1016/j.scib.2021.02.019>
5. Z. Chen, Y. Zhang, B. Zhu, Y. Wu, X. Du et al., Laser-sculptured hierarchical spinous structures for ultra-high-sensitivity iontronic sensors with a broad operation range. ACS Appl. Mater. Interfaces **14**(17), 19672–19682 (2022). <https://doi.org/10.1021/acsami.2c01356>
6. P. Wei, X. Guo, X. Qiu, D. Yu, Flexible capacitive pressure sensor with sensitivity and linear measuring range enhanced based on porous composite of carbon conductive paste and polydimethylsiloxane. Nanotechnology **30**(45), 455501 (2019). <https://doi.org/10.1088/1361-6528/ab3695>
7. Y. Xiao, Y. Duan, N. Li, L. Wu, B. Meng et al., Multilayer double-sided microstructured flexible iontronic pressure sensor with a record-wide linear working range. ACS Sens. **6**(5), 1785–1795 (2021). <https://doi.org/10.1021/acssensors.0c02547>
8. N. Bai, L. Wang, Y. Xue, Y. Wang, X. Hou et al., Graded interlocks for iontronic pressure sensors with high sensitivity and high linearity over a broad range. ACS Nano **16**(3), 4338–4347 (2022). <https://doi.org/10.1021/acsnano.1c10535>
9. H. Xu, L. Gao, H. Zhao, H. Huang, Y. Wang et al., Stretchable and anti-impact iontronic pressure sensor with an ultrabroad linear range for biophysical monitoring and deep learning-aided knee rehabilitation. Microsyst. Nanoeng. **7**, 92 (2021). <https://doi.org/10.1038/s41378-021-00318-2>
10. T. Yu, D. Zhang, Y. Wu, S. Guo, F. Lei et al., Graphene foam pressure sensor based on fractal electrode with high sensitivity and wide linear range. Carbon **182**, 497–505 (2021). <https://doi.org/10.1016/j.carbon.2021.06.049>
